# Trends in body-mass index and obesity in urban and rural China: findings from consecutive nationally representative surveys during 2004-2018

**DOI:** 10.1016/S0140-6736(21)00798-4

**Published:** 2021-07-03

**Authors:** Limin Wang, Bin Zhou, Zhenping Zhao, Ling Yang, Mei Zhang, Yong Jiang, Yichong Li, Maigeng Zhou, Linhong Wang, Zhengjing Huang, Xiao Zhang, Liyun Zhao, Dongmei Yu, Chun Li, Majid Ezzati, Zhengming Chen, Jing Wu, Gangqiang Ding, Xinhua Li

**Affiliations:** 1 National Center for Chronic and Non-communicable Disease Control and Prevention, Chinese Center for Disease Control and Prevention, Beijing, China; 2 National Institute for Nutrition and Health, Chinese Center for Disease Control and Prevention, Beijing, China; 3 MRC Centre for Environment and Health & Abdul Latif Jameel Institute for Disease and Emergency Analytics, Department of Epidemiology and Biostatistics, School of Public Health, Imperial College London, London, UK; 4 Clinical Trial Service Unit & Epidemiological Studies Unit, Nuffield Department of Population Health, University of Oxford, Oxford, UK; 5 National Clinical Research Center for Neurological Diseases, Beijing Tiantan Hospital, Capital Medical University, Beijing, China; 6 Fuwai Hospital Chinese Academy of Medical Sciences, Shenzhen, Guangdong, China; 7 People’s Medical Publishing House Co.LTD, Beijing, China

## Abstract

**Background:**

In China, mean body-mass index (BMI) and obesity in adult population have increased steadily since the early 1980s. However, there is no reliable assessment of their recent trends, nationally, regionally, or in certain population subgroups.

**Methods:**

Between 2004 and 2018 six nationally representative surveys in China included 645,223 adults aged 18-69 years, with measured height and weight. The mean BMI and prevalence of obesity (BMI ≥30 kg/m^2^) were calculated and their time trends compared by sex, age, urban-rural locality, geographical regions, and socioeconomic status.

**Findings:**

Overall, standardised mean BMI increased from 22.7 (95% CI: 22.5-22.9) in 2004 to 24.4 (24.3-24.6) kg/m^2^ in 2018 and obesity prevalence from 3.1% (2.5-3.7) to 8.1% (7.6-8.7), respectively. During 2010-2018, mean BMI rose by 0.09 (0.06-0.11) kg/m^2^ annually, which was half of that during 2004-2010 (0.17 [0.12-0.22]). Similarly, the annual increase in obesity prevalence was somewhat smaller after 2010 than before 2010 (6.0% [4.4-7.6] vs 8.7% [4.9-12.8]; p=0.13). Since 2010, the rise in mean BMI and obesity prevalence slowed down substantially in urban men and women, and moderately in rural men, but continued steadily in rural women. By 2018, mean BMI was higher in rural than urban women (24.3 vs 23.9 kg/m^2^; p=0.0045) but remained lower in rural than urban men (24.5 vs 25.1 kg/m^2^; p=0.0007). Across all six surveys, mean BMI was persistently lower in women with higher than lower education, but the converse was true among men. In China, about 85 million (48 million men, 37 million women) adults aged 18-69 were obese in 2018.

**Interpretation:**

In China, the rise in mean BMI among adult population appeared to have slowed down in last decade. However, there were divergent trends by sex, geographical areas and socioeconomic status, highlighting the need for more targeted approach to prevent further rise in obesity in general population.

**Funding:**

China National Key Research and Development Program, China National Key Project of Public Health Program, and Youth Scientific Research Foundation of NCNCD, China CDC

## Introduction

Obesity is a major risk factor for cardiometabolic diseases, renal disease and many cancers,^1–5^ affecting 670 million adults worldwide in 2016.^6^ Globally, the prevalence of obesity in adults has nearly tripled since 1975; in some middle-income countries, mean BMI has increased by 5 kg/m^2^ and prevalence of obesity by >10 folds.^6^ In China, mean BMI and obesity started to increase steadily since the 1980s following a rapid economic development,^7,8^ but they remained, at least by 2010, well below the levels seen in most other middle- and high-income countries. Despite this, a few national programmes targeting obesity and NCD prevention have been rolled out in China since 2010, including the National Demonstration Areas for Comprehensive Prevention and Control of NCDs and the China Healthy Lifestyle for All Initiative.^9,10^ In 2013 the World Health Assembly also set an ambitious global target for control of non-communicable diseases, including to halt, by 2025, the rise in the prevalence of obesity compared to 2010.^11^ Careful monitoring of long-term trends in BMI and obesity in general populations is essential to evaluate the likely success of various national and international initiatives and inform the development of country-specific health policies.

A few studies have previously reported on the rising trend in BMI and obesity prevalence in China, but they mainly used national survey data collected before 2013,^12–19^ including some global pooling studies,^6,20^ and tended to define obesity differently from those used in most international studies.^21^ The lack of reliable evidence on China’s recent trends in mean BMI and obesity both nationally and regionally, or by urban-rural locality which would reflect different stages of socio-economic development, led to substantial uncertainty over whether the rise in BMI will continue persistently. Moreover, important questions also persisted on whether the rising trends in BMI may vary significantly between men and women, urban and rural areas, or by socioeconomic status such as education, which could inform more targeted intervention measures.

To address the evidence gap, we presented detailed analyses of relevant data from six consecutive nationally representative health surveys conducted between 2004 and 2018. The main aims of the study were to: 1) examine the long-term and recent trends in mean BMI and prevalence of obesity among Chinese adults from 2004 to 2018, with specific emphasis on changes before and after 2010 (when various national NCD prevention programs were initiated); 2) assess how the trends may have varied by gender, age, urban-rural locality, and certain socioeconomic status; 3) estimate the number of people who were obese in 2018 versus 2004.

## Methods

### Survey design and populations

We used data from China Chronic Disease and Risk Factors Surveillance (CCDRFS) programme, which was established in 2004 with the aim to provide periodic nationwide data on the prevalence of major chronic diseases and the associated behavioural and metabolic risk factors in the general population. The details of the design, objectives and survey methods of CCDRFS have been described elsewhere,^22–24^ and summarised in the Appendix. In brief, the CCDRFS was incorporated into China’s Disease Surveillance Point (DSP) system, which now covers 324 million people (~24% of total population in China) across all 31 provinces, autonomous regions, and municipalities.^25,26^ Each DSP area covers a rural county or urban district which was selected using multi-stage stratified cluster sampling scheme.

**Figure 1:**
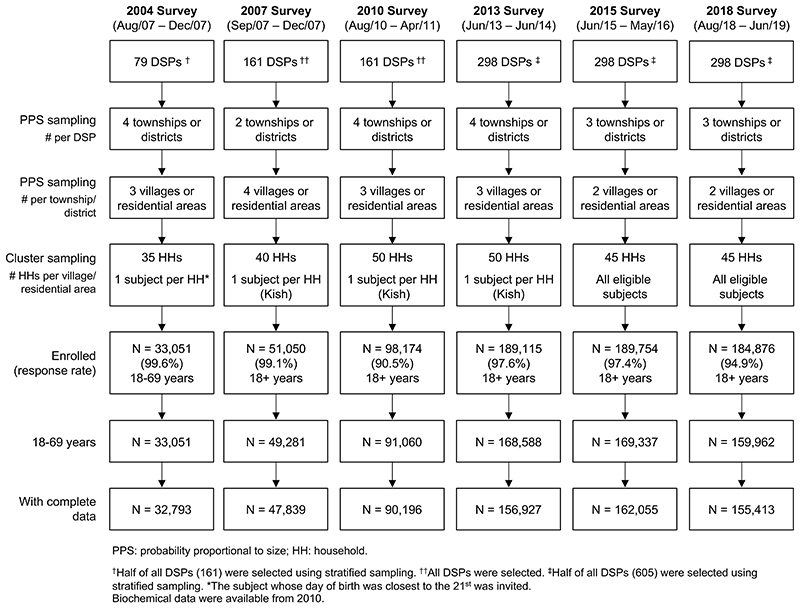
Flow diagram of study design and sampling procedure of CCDRFS 2004-2018.

For each CCDRFS survey, the participants were selected using multi-stage stratified cluster sampling within DSPs (Figure 1,and Appendix for further details). Across all six surveys, a total of 776,571 individuals were invited, and 746,020 (96.1%) participated, including 33,051 (response rate 99.6%) in 2004, 51,050 (99.1%) in 2007, 98,174 (90.5%) in 2010, 189,115 (97.6%) in 2013, 189,754 (97.4%) in 2015 and 184,876 (94.9%) in 2018. The study protocols for each survey were approved by the relevant national and regional ethical review committees. All participants provided written informed consent.

### Data collection

In each survey, trained health workers collected detailed questionnaire information on demographic and socioeconomic information, and undertook a range of physical measurements including standing height and body weight. In 2004 and 2007, height was measured using wall-mounted stature meters; since 2010, height was measured using mechanical anthropometry stadiometers (TGZ, Bengbu Equipment Inc., China). In 2004, weight was measured using metal body analog weighing scales; since 2007, weight was measured using the same model of electronic body scales, which were calibrated on a regular basis according to standard protocol (see Appendix).

### Statistical analysis

Overall 74,741 participants aged <18 or >69 years were excluded because these age ranges were not considered in the 2004 survey. We also excluded 26,056 (3.9%) participants with incomplete demographic or height/weight data across different surveys. After these exclusions, a total of 645,223 participants aged 18-69 years remained for the present analyses (Figure 1).

BMI was calculated as the weight in kilogram divided by the square of standing height in meter. We used standard WHO criteria to define overweight (BMI ≥25 kg/m^2^) and obesity (BMI ≥30 kg/m^2^). The mean BMI and prevalence of obesity in each survey were calculated after incorporating stratification, clustering and sample weights. To account for natural changes in population structures, multi-stage sampling design, non-response, and post-stratification, standard sample weights were constructed based on 2010 China census population (also see Appendix).

For mean BMI and prevalence of obesity, calculations were also performed seperatedly by sex (men/women), locality of residence (urban/rural), age (five groups), education (four groups), occupation (five groups), and geographical region (seven groups). When results were not stratified by age, standardised mean and prevalence were calculated by averaging the sex- and age- specific mean and prevalence in each survey weighted by the 2010 China census population. We used the *proc surveymeans* procedure in SAS to estimate standard erros and 95% confidence intervals, using Taylor series linearisations with finite population correction. All analyses accounted for complex sample design including clustering, stratification, and sample weights.

We further compared trends before and after 2010 to assess potential impacts on trends in BMI and obesity of new national programmes related to obesity and NCD prevention introduced around 2010. The annual changes in mean BMI were calculated as the absolute difference in mean BMI between the start and end years divided by total number of years covered. The annual relative changes in overweight and obesity prevalence were calculated as the difference in prevalence between the the start and end years divided by the prevalence in the start year annualised by accounting for compounding (see Appendix). We performed the Student’s t-test for trends for each characteristics in Table 1 and Welch’s t-test for comparisons of mean between groups and over time, and to compare trends in mean BMI before and after 2010; we used a simulation-based method^27^ for prevalence of obesity and overweight (see Appendix). We did not adjust for possible multiple comparisons involved in our analysis. All analyses were done in SAS 9.4 (SAS Institute Inc., Cary, North Carolina, USA) and R version 3.6.0 (R Foundation for Statistical Computing, Vienna, Austria).

### Role of funding source

The funders of the study had no role in the design or conduct of the study, including data collection, management, analysis, or interpretation of the results; preparation, review, or approval of the manuscript; or the decision to submit the manuscript for publication.

## Results

From 2004 to 2018, the number of participants included in each survey increased from 32,793 to 155,413, with increasing proportions of older people (mean age increased from 44 years to 52 years; p-value for trend=0.0070) and urban residents (38% to 46%; p=0.0012) (Table 1). Moreover, a higher proportion of participants performed non-manual works (20% to 38%; p=0.0269), while the converse was true for agriculture-related work (57% to 44%; p=0.0161). However, there was little change in the distribution of sample across regions and education levels over the study period.

Overall the standardised mean BMI levels rose from 22.7 (95% CI: 22.5-22.9) kg/m^2^ in 2004 to 24.4 (24.3-24.6) kg/m^2^ in 2018 (Appendix Table 1), driven expectedly by increase in body weight rather than changes in height (Appendix Figure 1). From 2004 to 2010 the mean BMI rose by 0.17 (0.12-0.22) kg/m^2^ annually, which was twice the annual increase (0.09 [0.06-0.11] kg/m^2^) observed between 2010 and 2018 (Table 2). Consistent with increase in mean BMI and prevalence of obesity (i.e. a shift in the BMI distribution to the right), we found that BMI distributions became wider from 2004 to 2018 (Appendix Figure 2), as did their corresponding SDs, but the increase in SD was smaller after 2010 especially among men (Appendix Figure 3). In 2004, men had lower mean BMI and prevalence of obesity than women, but by 2018 this had been totally reversed because on average BMI rose more rapidly in men than in women between 2004 and 2018 (annual change of 0.16 [0.14-0.18] vs 0.09 [0.07-0.11] kg/m^2^; p<0.0001) (Table 2 and Appendix Table 1).

In 2018, the standardised prevalence of obesity was 8.1% (7.6-8.7), more than twice as high as in 2004 (3.1% [2.5-3.7]) (Appendix Table 1). In absolute number, it was estimated that 85 (70-100) million adults (48 [39-57] million men and 37 [31-43] million women) aged 18-69 years in China were obese in 2018, which was three times as many as in 2004 (total 28 million; 12 million men and 16 million women). Again, the rise in obesity over the study period was somewhat smaller after 2010 (6.0% [4.4-7.6] annual relative increase) compared to before 2010 (8.7% [4.9-12.8]) (Table 2). The slowdown in the rise of obesity prevalence was evident across all regions except in Southern China, although it was distributed unevenly across age groups and education levels (Appendix Table 2). Likewise, for the prevalence of overweight the annual increase was about half as fast after 2010 as before 2010 (2.7% [2.0-3.4] vs 6.4% [4.6-8.4]; p=0.0007) (Table 2).

**Figure 2:**
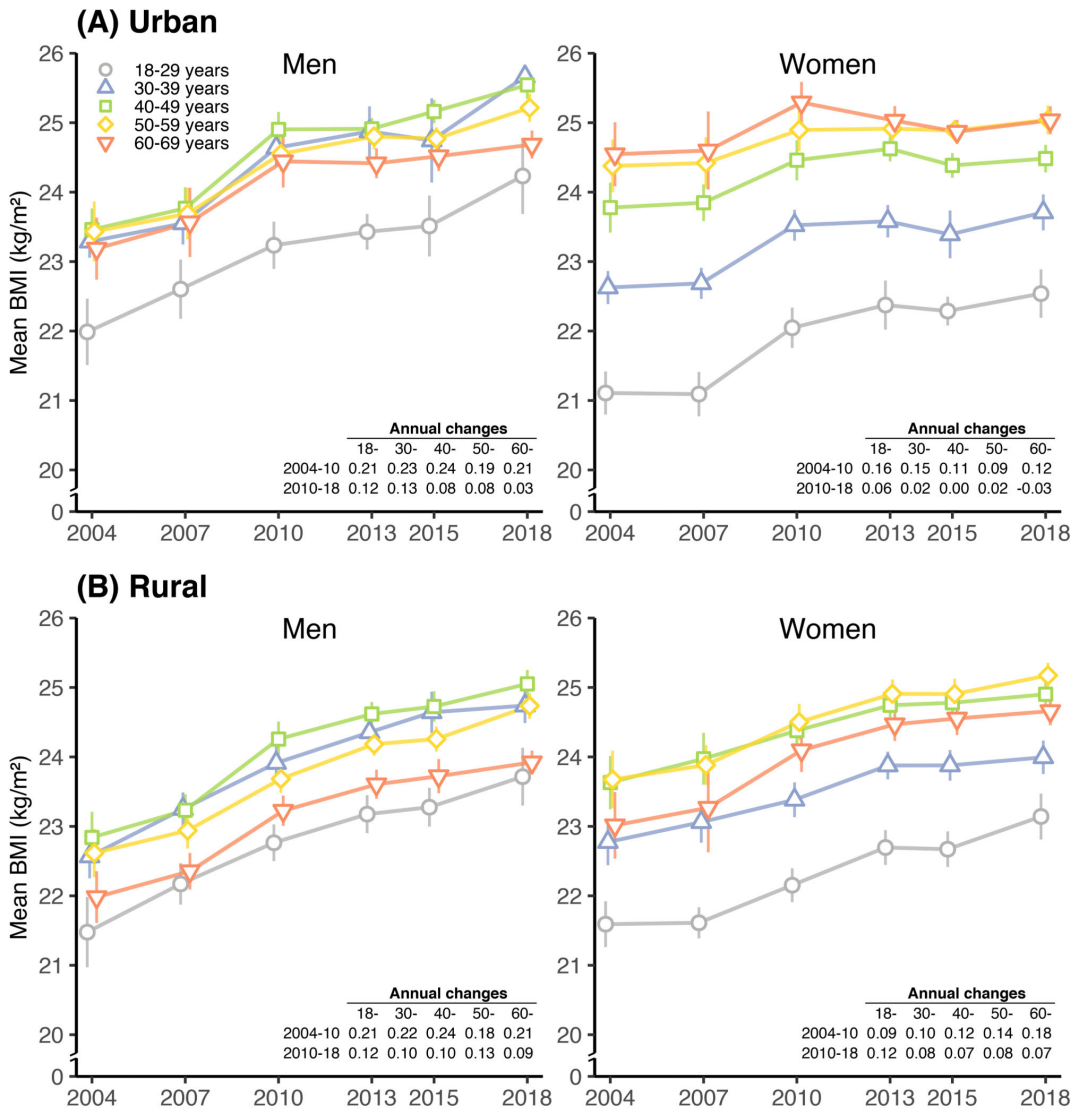
Trends in standardised mean BMI in (A) urban and (B) rural areas from 2004 to 2018, by sex and age group. Lines show the 95% CIs of the mean or prevalence. Results are standardised to the 2010 China census population. Annual change in mean BMI and annual relative change in prevalence of obesity and overweight before and after 2010 are listed in the table in each panel.

The trends in mean BMI and obesity prevalence were largely similar across different age groups for men and women in both urban and rural areas (Figure 2 and Appendix Figure 4), except that in urban areas the pace of rise in mean BMI and obesity slowed down more rapidly in older than younger women (Appendix Table 2). In both sexes the lowest mean BMI was in those aged 18-29 years (Figure 2), while the lowest obesity prevalence was seen at the youngest age group in women but at the oldest age group in men (Appendix Figure 4). Overall among women, mean BMI and obesity prevalence generally increased with age until about 50 years of age, with little further change afterwards; among men there was a more complex pattern, with middle-aged men (30-49 years) having the highest mean BMI and prevalence of obesity at each survey (Figure 2 and Appendix Figure 4).

**Figure 3:**
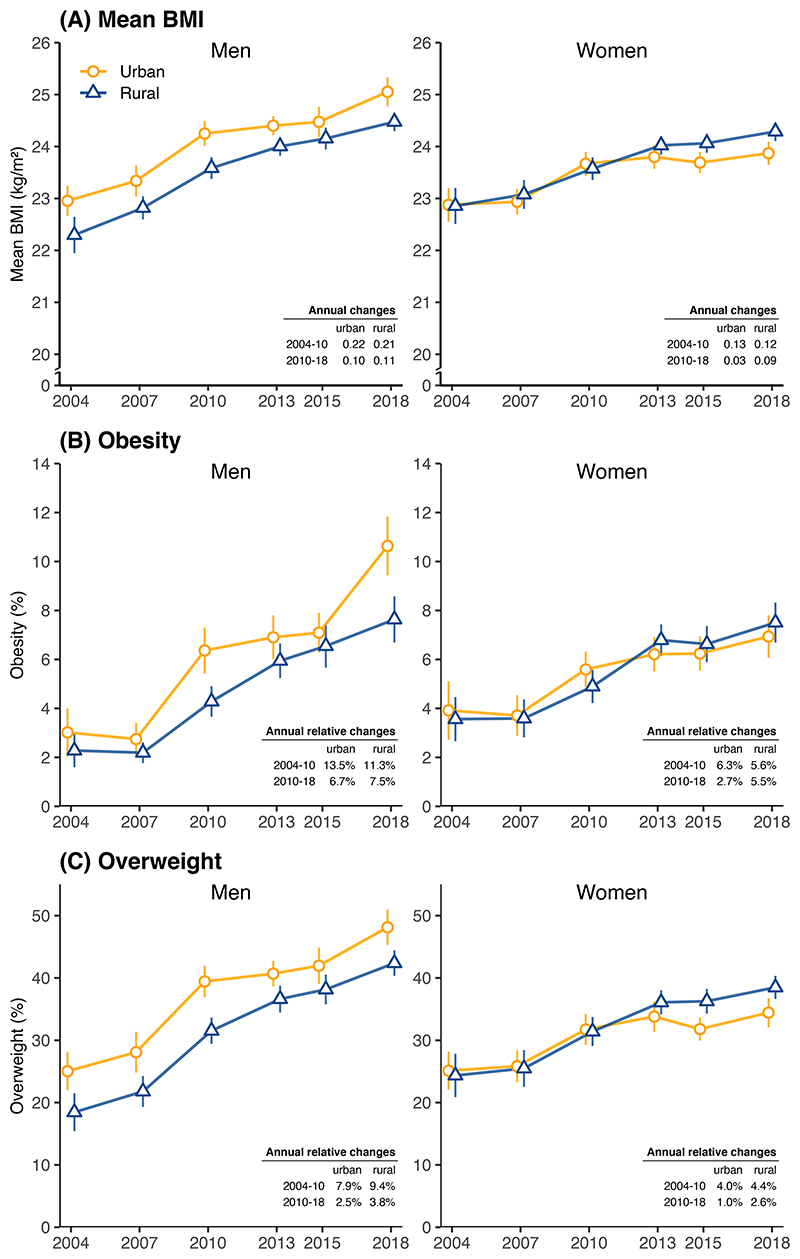
Trends in standardised (A) mean BMI, (B) prevalence of obesity and (C) prevalence of overweight from 2004 to 2018, by sex and urban-rural locality. See Appendix Table 1 for numerical results. Conventions are the same as in Figure 2.

From 2004 to 2010, the mean BMI and prevalence of obesity rose similarly in urban and rural areas, by about 0.17 kg/m^2^ annually for mean BMI and 8-10% annually for obesity (Figure 3 and Table 2). Over this period, urban men and women had higher mean BMI and prevalence of obesity than their rural counterparts (Appendix Table 1). After 2010, however, the trends diverged between urban and rural women. Among urban women, the rise in mean BMI nearly stopped (increasing by 0.03 [-0.01, 0.07] kg/m^2^ annually), whereas the rise among rural women continued at 0.09 (0.05-0.12) kg/m^2^ annually (Table 2). Among urban and rural men, however, the rise in mean BMI continued, albeit at a slower rate compared to before 2010 (~0.1 kg/m^2^ annually). For obesity (as well as overweight), the annual rate of increase was halved after 2010 compared with that before 2010 in both men (6.7% [4.2-9.2] vs 13.5% [7.2-20.9]; p=0.0400) and women (2.7% [0.4-5.1] vs 6.3% [0.8-12.8]; p=0.1546). However, in rural areas, the situation appeared different after 2010, with the rise in obesity only moderately slowed down in men (11.3% to 7.5%; p=0.1600) but continued at a similar pace in women (5.6% to 5.5%; p=0.4983) (Table 2). Consequently, by 2018, rural women had higher mean BMI and obesity prevalence than their urban counterparts (p=0.0045) (Figure 3 and Appendix Table 1); among men the similar urban-rural switchover has not yet taken place, partly because a large increase in obesity among urban men between 2015 and 2018. We did not observe divergent trends in the SD of BMI between urban and rural women, suggesting that the larger rise in obesity in rural women after 2010 was a population wide phenomenon rather than a change among high-BMI individuals.

From 2004 to 2018, the rise in mean BMI was larger among men and women with lower education than those with higher education. However, there was no clear educational pattern for the rise in prevalence of obesity. The slowdown or plateauing in mean BMI and urban obesity since 2010 happened across all education groups, but the rise in rural obesity accelerated among women with secondary or higher levels of education (Appendix Table 2).

**Figure 4:**
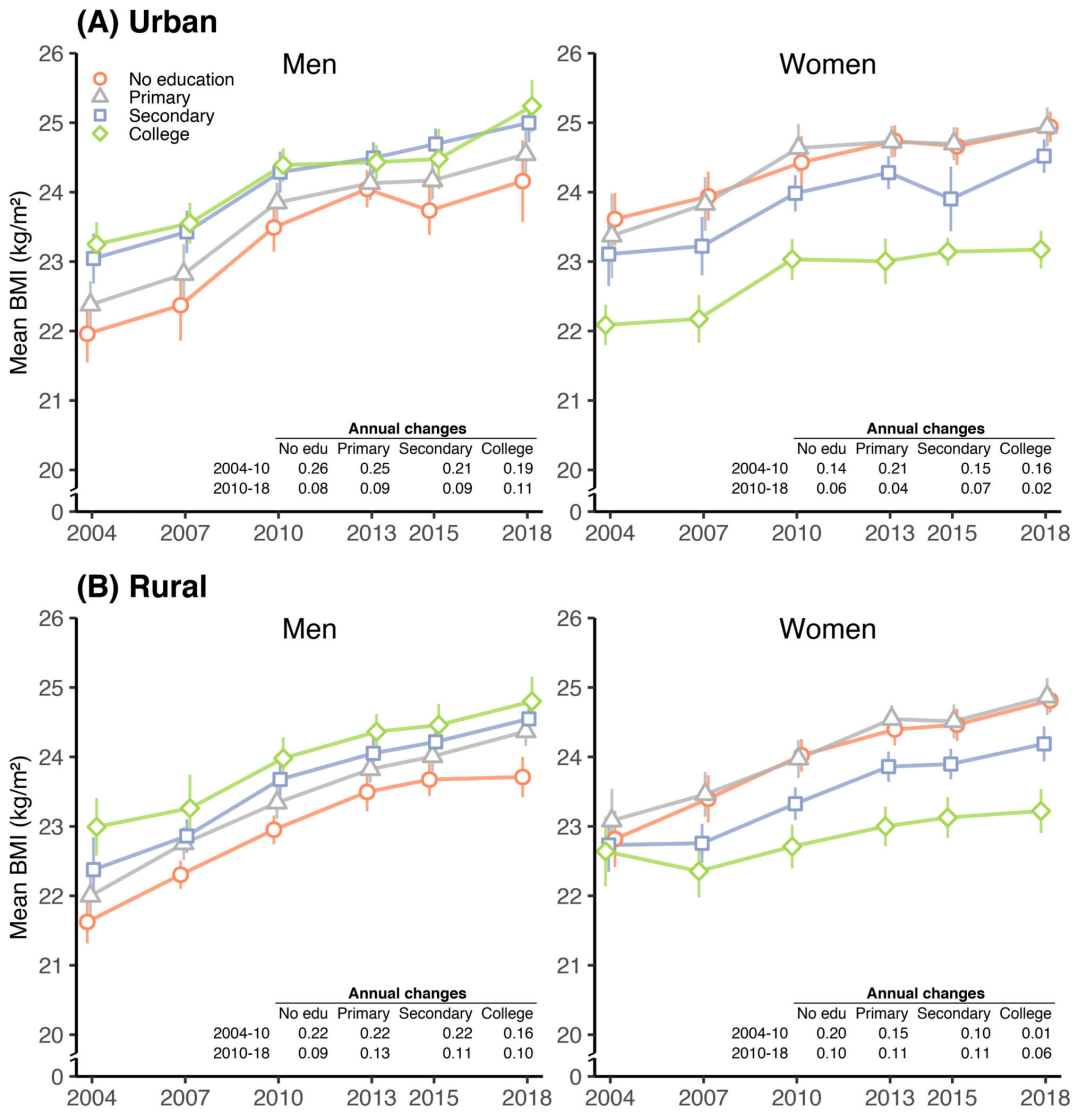
Trends in standardised mean BMI in (A) urban and (B) rural areas from 2004 to 2018, by sex and education. Conventions are the same as in Figure 2.

In 2018, urban and rural men with the highest education had a mean BMI ~1.1 kg/m^2^ higher, and an obesity prevalence more than 100% higher, than those with the lowest education (Figure 4 and Appendix Figure 5). By contrast, urban and rural women with the highest education had lower mean BMI (by 1.6-1.8 kg/m^2^) and obesity prevalence (by 20-30%) than those with the lowest education.

The trends in mean BMI and obesity prevalence were similar across different occupation groups (Appendix Figures 6 and 7). In both urban and rural areas, men with non-manual work had seen a large rise in obesity prevalence between 2015 and 2018, but this was not evident among their female counterparts. Men working in agriculture related profession had the lowest obesity but women working in agriculture had similar levels of obesity to those working in other professions.

Trends in mean BMI and obesity prevalence were largely consistent across 7 geographical regions of China (Appendix Figures 8 and 9). A persistent north-south difference in both mean BMI and obesity prevalence was observed in men and women and in urban and rural areas. Overall, people living in Northern China had a mean BMI more than 2 kg/m^2^ higher than their counterparts in Southern China. Likewise, there was >3-fold difference in obesity prevalence between Northen and Southern China.

In sensitivity analyses involving just 60 DSPs that were covered in all six surveys with no changes in geographical boundaries, we observed similar results as in the main analysis, including slowdown in urban BMI and obesity, with the exception of urban female obesity which continued to rise at a similar pace since 2010 compared to before 2010 in these 60 DSPs (Appendix Figure 10). Likewise, when using Chinese cut-off points to define obesity (≥28 kg/m^2^) and overweight (≥24 kg/m^2^), the results were generally similar to that based on the WHO definition in the main analyses (Appendix Tables 3 and 4).

## Discussion

Using large-scale data from six nationally representative population surveys covering a period from 2004 to 2018, the present study showed that, while the prevalence of obesity has more than doubled and total number of adults who were obese more than tripled since 2004, there were divergent trends in urban and rural areas, especially among women. In urban China, the rise in adult BMI and obesity may be plateauing in women and slowing down in men, but in rural China they continue to rise steadily, especially amonng women. The divergent trends led to higher mean BMI and obesity prevalence in rural than in urban women in 2018, with narrowing urban-rural difference among men as well. Across all six surveys, the mean BMI was persistently lower in those with higher than lower education among women, but the converse was true among men, and geographically there were persistent north-south gradients in mean BMI and obesity prevalence. It was estimated that in 2018 a total of 85 million (48 million men, 37 million women) adults aged 18-69 years were obese in China, representing >3-fold increase in about 15 years.

Previous studies predicted a massive epidemic of obesity in China, based mainly on experiences of other low- and middle-income countries (LMICs).^6,28^ For example, in Mexico, Brazil and Malaysia, mean BMI and obesity increased progressively to eventually surpasse the levels seen in most high-income countries.^29–31^ Different from these countries, China’s recent trends in BMI, and the plateauing of urban female BMI, as we found, are more in line with the experiences of high-income countries in East Asia (e.g., Japan and South Korea) and Western Europe (e.g., France).^6,20,32–34^ As in the present study, many LMICs and most high-income countries also found BMI in rural populations to be rising faster than in urban areas; however, the urban-rural switchover of female BMI, as we reported in China, was only observed in high-income countries but not yet evident in most other LMICs.^20^ In many LMICs, including those in South Asia and Southeast Asia, BMI rose faster among women than men,^20^ in contrast to our findings in China.

A few nationwide studies have previously reported on the levels and trends in mean BMI and obesity prevalence in China. Both our early (i.e. pre-2010) and recent results (post-2010) were generally consistent with relevant findings in other studies at particular time point,^35–41^ and a rising trend in BMI in general adult populations up to 2013.^12–19^ Existing evidence on national trends beyond 2013 in China was mainly based on statistical models in global studies,^6,20^ or from studies that used an alternative cut-off to define obesity which limited their comparability. A nationwide study, involving non-random sampling methods reported steady rise in mean BMI and prevalence of obesity (defined as BMI ≥27.5 kg/m^2^) among adults aged 18-80 years during 1993-2015.^21^ In that study, the annual increase in mean BMI (0.17 kg/m^2^) and obesity prevalence (12%) were similar to our results during 2004-2010. In another study of >15 million married couples aged up to 49 years in rural China who were planning pregnancy,^42,43^ the reported prevalence of obesity (defined as BMI ≥28 kg/m^2^) at around 2014 was lower than our findings using the same definition for both men (7% vs 14%) and women (5% vs 14%). The only study that reported trends post 2015 used data from two national surveys involving 426,000 adults aged ≥19 years from 31 provinces in China during 2013-2018.^44^ In that study, the reported annual increases in prevalence of overweight (6%) and obesity (12%), using WHO definition, were greater than our study (2% and 5%, respecitively) during the same period. On the other hand, the obesity prevalence reported in that study for 2018 (3.7%) was noticeably lower than our results (8.1%), which was, as acknowledged in the paper, lower than other nationwide surveys, and could be due to inappropriate sampling methods used (e.g., 26% of participants were unemployed).^44^ By analysing data from six consecutive surveys during 2004-2018, the present study provided robust new evidence about evolving epidemic of obesity in China, not only overall but also regionally and by urban-rural locality.

Although a few studies in China have previously assessed the urban and rural trends seperatly, they did not identify the emerging divergent trends as we did, mainly because of the time period covered.^14,17^ The drivers behind the increase and the divergent trends in urban and rural China are complex, and may reflect differences in stages of economic development, changing levels and patterns of physical activity, and increase in total calorie intake and consumption of animal-based food.^45–47^ In China, despite an overall increase in leisure-time physical activity since 2000,^48^ the total physical activity has declined sharply from 1991 to 2009, especially in rural areas.^49^ Our study also showed that the proportion of people working in agricultural and manual occupations decreased from 60% in 2004 to 38% in 2018. Even among those in agricultural jobs, increased use of machines may also have greatly reduced the level of physical activity involved. Throughout the study period, although income has been increasing steadily in both urban and rural China,^50^ rural residents may have used disproportionally more of their increased income on foods than did urban residents, having initially had lower incomes. With higher income and a massive rise in packaged and processed foods in both rural and urban areas,^51^ the composition of Chinese diet is changing, generally towards higher intake of fat and animal product.^52–54^ There is evidence that better educated people and those in more urbanised areas had higher score in ‘healthy eating’,^55^ while economically more developed urban area had seen greater improvement in diet quality in recent decades.^56^ Data from Food and Agriculture Organization also showed large increase in daily calorie supply and the proportion from animal source foods and sugar in China during this period.^57,58^

The contrasting education gradients in BMI and obesity between men and women observed in our study are consistent with two smaller studies in China using non-nationwide samples in multiple provinces.^59,60^ Previous studies in high- or upper-middle-income countries also reported similar findings among women, but mixed results amon men,^61^ with little high-quality data from other LMICs.^62^ Our study now provided reliable evidence about the contrasting associtatoins of education with BMI and obesity between men and women, but further studies are needed to elucidate the likely contributing factors.

Apart from large sample size and nationally representative samples covering periods from 2004 to 2018, the main strengths of our study included high response rates, use of largely consistent protocols over time, and ability to assess the trend by sex, urban-rural locality, geographical regions and certain socioeconomic status. However, our study also has limitations. First, the surveys were not conducted annually, which could have allowed more detailed and quantitative analysis of the plateauing or slowdown in trends, but this will be monitored and assessed in future surveys. Second, the number of DSPs covered across different surveys, hence sampling scheme and study size, changed over time, potentially introducing bias in the observed trends. Nevertheless, in the sensitivty analyses of 60 areas that were included in all six surveys, the results were similar to that in our main analyses. Third, although the instruments used for measuring height and weight have not changed since 2007, a different model was used in 2004, which may affect the comparability of its data with later rounds. Despite this, weight can be measured reliably by different devices and there were regular calibrations in field surveys to ensure data quality. Fourth, our sample had a smaller proportion of men (42-47%) compared with the 2010 census population (50%). This is because, as commonly done in household surveys, our surveys only sampled individuals who had been living in the same address for at least 6 months in the past 12 months. Therefore, people who worked away from their home address were not eligible to participate, which would disproportionally affect men compared with women, and affect the representativness of the study populations. To minimise the likely impact, we used the sex-specific 2010 census population to estimate the overall results.

In summary, the present study provided robust new evidence about the evolving epidemic of obesity among adult populations in China. While the rise in mean BMI among adult population appeared to have slowed down in last decade, there were divergent trends between urban and rural areas, contrasting associations of education with adiposity in men and women, and large variations in obesity prevalence across different regions. As China continues to modernise and becomes more urbanised, obesity and the associated health burdens (e.g., hypertension and diabetes) are likely to become more and more a rural issue in the coming decades. Our study findings highlighted the need to continue to monitor the longer term trends nationally and regionally, and for better understanding of the factors underlying these trends in order to develop more targeted and effective prevention strategies in both urban and rural China.^63^

## Supplementary Material

Appendix

## Figures and Tables

**Table 1:** Characteristics of study participants in each survey

Characteristics		2004 (N=32,793)	2007 (N=47,839)	2010 (N=90,196)	2013 (N=156,927)	2015 (N=162,055)	2018 (N=155,413)	p for trend
Men, %		44.3	47.3	45.6	42.3	46.0	43.7	0.5249
Age group, %	18-29 years	11.9	13.1	16.1	8.9	9.8	6.2	0.1269
	30-39 years	26.3	24.1	19.7	14.4	13.4	11.6	0.0004
	40-49 years	27.4	25.4	27.3	27.5	24.7	20.9	0.1251
	50-59 years	21.6	23.2	22.9	27.9	27.3	30.2	0.0042
	60-69 years	12.7	14.1	14.0	21.2	24.8	31.2	0.0048
Mean age (SD), years		44.1 (12.2)	45.0 (12.7)	44.3 (13.1)	48.9 (12.2)	49.4 (12.6)	51.9 (12.0)	0.0070
Urban, %		37.5	38.9	39.2	41.5	42.7	45.5	0.0012
Region, %	Central	10.8	13.2	13.5	12.4	12.3	12.8	0.4669
	East	23.1	24.9	24.1	26.0	25.3	24.8	0.1694
	North	12.9	14.2	14.2	14.7	14.3	14.7	0.0477
	Northeast	12.6	10.7	11.2	9.7	9.6	9.5	0.0139
	Northwest	15.2	12.3	12.3	13.0	13.2	13.2	0.4855
	South	8.9	8.8	8.5	9.8	9.9	10.2	0.0372
	Southwest	16.5	15.9	16.3	14.4	15.3	14.8	0.0592
Education, %	No education	18.6	24.9	21.6	25.2	26.1	26.3	0.0539
	Primary	29.1	19.6	19.3	19.9	20.1	19.5	0.1549
	Secondary	32.6	32.4	33.4	33.3	32.5	32.5	0.9848
	Collge	19.6	23.1	25.7	21.6	21.3	21.7	0.9206
Occupation, %	Agriculture related	56.6	48.9	47.7	47.3	45.8	44.3	0.0161
	Other manual work	5.7	6.9	4.1	4.8	4.1	3.5	0.0580
	Non-manual work	19.8	27.0	38.0	38.2	38.4	38.0	0.0269
	Not working	10.8	9.4	3.5	2.9	4.2	4.9	0.0768
	Retired	7.1	7.8	6.7	6.8	7.4	9.4	0.2747

**Table 2:** Annual change in standardised mean BMI and prevalence of obesity and overweight during 2004-2010 and 2010-2018

	Men			Women			Both		
	2004-2010	2010-2018	P*	2004-2010	2010-2018	p*	2004-2010	2010-2018	p*
**Change (& 95% CI) in mean BMI** (kg/m^2^)									
Overall	0.22 (0.17, 0.27)	0.11 (0.08, 0.14)	0.0014	0.12 (0.07, 0.17)	0.06 (0.03, 0.09)	0.0299	0.17 (0.12, 0.22)	0.09 (0.06, 0.11)	0.0044
Urban	0.22 (0.15, 0.28)	0.10 (0.05, 0.15)	0.0067	0.13 (0.07, 0.20)	0.03 (-0.01, 0.07)	0.0102	0.17 (0.12, 0.23)	0.06 (0.02, 0.10)	0.0046
Rural	0.21 (0.15, 0.28)	0.11 (0.08, 0.15)	0.0096	0.12 (0.05, 0.19)	0.09 (0.05, 0.12)	0.2507	0.17 (0.10, 0.23)	0.10 (0.07, 0.13)	0.0568
**% change (& 95% CI) in obesity prevalence**									
Overall	12.2 (7.8, 17.1)	7.5 (5.7, 9.4)	0.0464	5.8 (2.1, 9.8)	4.4 (2.7, 6.1)	0.2796	8.7 (4.9, 12.8)	6.0 (4.4, 7.6)	0.1346
Urban	13.5 (7.2, 20.9)	6.7 (4.2, 9.2)	0.0400	6.3 (0.8, 12.8)	2.7 (0.4, 5.1)	0.1546	9.7 (4.2, 16.1)	5.0 (2.8, 7.2)	0.0831
Rural	11.3 (5.5, 18.1)	7.5 (5.0, 10.1)	0.1600	5.6 (0.7, 11.0)	5.5 (3.2, 7.9)	0.4983	8.0 (3.1, 13.5)	6.5 (4.4, 8.7)	0.3257
**% change (& 95% CI) in overweight prevalence**									
Overall	8.7 (6.6, 10.9)	3.4 (2.6, 4.2)	<0.0001	4.2 (2.3, 6.2)	1.9 (1.1, 2.8)	0.0313	6.4 (4.6, 8.4)	2.7 (2.0, 3.4)	0.0007
Urban	7.9 (5.5, 10.5)	2.5 (1.4, 3.6)	0.0002	4.0 (1.6, 6.6)	1.0 (-0.3, 2.3)	0.0357	6.1 (3.8, 8.5)	1.9 (0.8, 3.0)	0.0019
Rural	9.4 (6.3, 12.8)	3.8 (2.7, 4.8)	0.0009	4.4 (1.7, 7.3)	2.6 (1.5, 3.7)	0.1498	6.7 (4.0, 9.7)	3.2 (2.2, 4.2)	0.0168

* p-value for difference in annual changes for 2004-2010 versus 2010-2018.

## Data Availability

Individual participant data in our study will not be made available publicly. For further detailed data assess policy and procedure, please contact jianceshi@ncncd.chinacdc.cn.

## Research in Context

### Evidence before this study

We searched PubMed for studies on national trends in mean BMI and obesity in China, using the search terms ((obesity[MeSH Terms] OR (body mass index[MeSH Terms])) AND adult[MeSH Terms] AND China[MeSH Terms]) AND trend*[Title/Abstract]. We found several studies that reported a rise in mean BMI and obesity until 2013 in both urban and rural areas and across different education groups. Evidence on post-2013 trends in BMI and obesity in China was mainly based on studies in particular regions and from statistical models in global studies. One study reported steady rise in mean BMI and obesity (defined as BMI ≥27.5 kg/m^2^) among 18-80 year-old adults during 1993-2015. Another study found increasing trends in the prevalence of obesity (defined as BMI ≥28 kg/m^2^) among rural men agd 15-49 years during 2010-2014. The use of Chinese BMI cut-offs for obesity, which was proposed in the early 2000s, in these studies limited the ability to compare with data in China collected before 2004 or with data in other countries. The only recent study in China that classified obesity using conventional WHO definition covered only a five-year priod during 2013-2018 and found a rising trend in the prevalence; however, it did not examine long-term trends before 2013, nor trend in mean BMI. Moreover, it used an unconventioal sampling method, leading to an unexpected much lower obesity prevalence than seen in other nationwide surveys in China. To our knowledge, there are no national studies covering a prolonged time period until recent years in mean BMI or in the prevalence of obesity using internationally comparable definitions (BMI ≥30 kg/m^2^) in China.

### Added value of this study

The present study provides the most up-to-date analyses of the long-term and recent trends in mean BMI and obesity prevalence in China. By using six large national surveys during 2004-2018, we examined the trends both nationally and by sex, age, urban-rural locality, education, occupation, and geographical region. Our study showed that, different from previous predictions, the rise in mean BMI and obesity prevalence appeared to have slowed down in China after 2010 when a number of large nationwide prevention programmes targeting NCD or obesity were rolled out. However, there were divergent trends in urban and rural areas, particularly among women. Since 2010 the rise in mean BMI and obesity prevalence slowed down substantially in urban men and women, and moderately in rural men, but continued steadily in rural women. By 2018, mean BMI was higher in rural than urban women (24.3 vs 23.9 kg/m^2^; p=0.0045) but remained lower in rural than urban men (24.5 vs 25.1 kg/m^2^; p=0.0007). Among women, the mean BMI was persistently lower in those with higher than lower education across all six surveys, but the converse was true among men. In China, 85 million (48 million men, 37 million women) adults aged 18-69 were obese in 2018, representing >3-fold increase since 2004.

### Implication of all the available evidence

Although the total number of adults who were obese reached 85 millioin in 2018, the rise in mean BMI and obesity appeared to be plateauing in urban Choina while in rural China they continue to rise, especially among adult women. In addition to divergent trends in urban and rural areas, there were also large disparities in BMI and obesity trend by sex, age, and socioeconomic status. While further monitoring is needed to assess the longer term trends and changing patterns, the present study findings highlight the pressing need for more targeted health policies to reduce further increase in obesity in the general population, with a gradual shift towards placing more emphasis on rural populations.
