# Supplementary material for: Trends in body-mass index and obesity in urban and rural China: findings from consecutive nationally representative surveys during 2004-2018
Source: Lancet. Author manuscript; Available in PMC 2024 Dec 9. (PMC7617101; doi:10.1016/S0140-6736(21)00798-4)
Supplement: Appendix [file EMS201458-supplement-Appendix.pdf]

THE LANCET

Supplementary appendix

This appendix formed part of the original submission and has been peer reviewed. We post it as supplied by the authors.

Supplement to: Wang L, Zhou B, Zhao Z, et al. Body-mass index and obesity in urban and rural China: findings from consecutive nationally representative surveys during 2004–18. *Lancet* 2021; **398:** 53–63.

Appendix

Trends in body-mass index and obesity in urban and rural China: findings from consecutive nationally representative surveys during 2004-2018, by Limin Wang, et al.

Table of contents

Appendix 1. Sample design of CCDRFS. **3**

Appendix 2. Data collection of CCDRFS. **6**

Appendix 3. Analysis plan. **7**

Appendix Figure 1. Trends in standardised mean height from 2004 to 2018, by sex and urban-rural locality. **10**

Appendix Figure 2. Change in BMI distribution from 2004 to 2018, by sex and urban-rural locality. **11**

Appendix Figure 3. Trends in standard deviation of BMI from 2004 to 2018, by sex and urban-rural locality. **12**

Appendix Figure 4. Trends in standardised prevalence of obesity from 2004 to 2018 in (A) urban and (B) rural areas, by sex and age group. **13**

Appendix Figure 5. Trends in standardised prevalence of obesity from 2004 to 2018 in (A) urban and (B) rural areas, by sex and education. **14**

Appendix Figure 6. Trends in standardised mean BMI from 2004 to 2018 in (A) urban and (B) rural areas, by sex and occupation. **15**

Appendix Figure 7. Trends in standardised prevalence of obesity from 2004 to 2018 in (A) urban and (B) rural areas, by sex and occupation. **16**

Appendix Figure 8. Trends in standardised mean BMI from 2004 to 2018 in (A) urban and (B) rural areas, by sex and region. **17**

Appendix Figure 9. Trends in standardised prevalence of obesity from 2004 to 2018 in (A) urban and (B) rural areas, by sex and region **18**

Appendix Figure 10. Trends in mean BMI and prevalence of obesity and overweight from 2004-2018, using data from DSPs that have been included in all survey rounds, by sex and urban-rural locality. **19**

Appendix Table 1. Standardised mean BMI, prevalence of obesity and prevalence of overweight. **20**

Appendix Table 2. Annual change in standardised mean BMI, prevalence of obesity and prevalence of overweight from 2004 to 2010 and from 2010 to 2018 in different age groups, education levels, occupation groups and regions, by sex. **22**

Appendix Table 3. Standardised prevalence of obesity and overweight defined by Chinese BMI cut-offs (obesity: BMI ≥28 and overweight: ≥24 kg/m^2). **31**

Appendix Table 4. Annual change in standardised prevalence of obesity and overweight defined by Chinese BMI cut-offs (obesity: BMI ≥28 and overweight: ≥24 kg/m^2) in 2004-2010 and in 2010-2018. .... **32**

Appendix 1. Sample design of CCDRFS.

Overview

The sample of CCDRFS was obtained from China's national Disease Surveillance Point (DSP) system.[1] One DSP unit covers a rural county or an urban district. The DSPs were established during early1980s, using multi-stage stratified sampling with probability proportional to size (PPS) to ensure representativeness of the national population of mainland China. Following further expansion and sampling enhancement, from the 2013 survey the selected DSPs were also representative at provincial level. CCDRFS is planned and administrated by the National Center for Chronic and Non-communicable Disease Control and Prevention (NCNCD) of the Chinese Center for Disease Control and Prevention (CDC).

Disease Surveillance Point (DSP)

The DSP system was piloted in 1978 and fully established in early 1980s covering initially 71 sites, expanding to 145 in 1989 and 161 DSPs in 2004 (covering a population of 73 million), in order to accommodate the societal and economic development during this period. For each expansion, the population characteristics of the selected DSPs were compared to the census population to ensure its nationally representativeness.[2,3] In 2013, the Chinese government combined the DSP system with the national vital registration system to form an integrated national mortality surveillance system, which increased the DSP areas from 161 to 605. The DSPs system currently covers 324 million Chinese adults (24% of all Chinese population) in the mainland of China.

Sampling procedure

Embedded within the DSP system, the CCDRFS used stratified multi-stage cluster sampling to generate a nationally representative sample for each survey.

In the first stage, half of the DSPs were selected as primary sampling units (PSUs) using stratified sampling (in the surveys of 2004, 2013, 2015 and 2018), or all DSPs were selected as PSUs (in the surveys of 2007 and 2010). Since the 2013 survey, following the expansion of the DSP system, 298 DSPs were selected from all 605 DSPs to generate a sample representative of both the national and provincial population in the 31 provinces of mainland China. The sampling of DSPs was done centrally by the NCNCD.

Then, within each selected DSP, the following steps were followed:

1. Townships (rural) or subdistricts (urban) were selected with PPS sampling in each selected DSP.
2. Within the selected townships and subdistricts, villages (rural) or residential areas (urban) were selected with PPS sampling.
3. Each selected village or residential area was divided into groups of about 50 households, based on existing villager/resident groups in the village or residential area. One group was selected with simple random sampling.
4. Within each selected household, in the 2004 survey, one eligible adult whose day of birth was closest to the 21st was invited. In the surveys of 2007, 2010, and 2013, the Kish method was used to select one eligible adult. In the 2015 and 2018 surveys, all eligible adults in the household were invited.

   Eligibility criteria include:

   a. Aged 18 years or older;

   b. Having lived in the address for more than 6 months in the past 12 months;
   c. Not pregnant;
   d. Not having a serious health condition or illness that prevents from participating, including intellectual disability or language disorder.
5. In a household, if the selected eligible adult (the surveys of 2004, 2007, 2010, and 2013), or more than half of the family's eligible members (the 2015 and 2018 surveys) refused to participate or were not reachable, a replacement household with similar sex and age structure was selected. In each round, <10% households were replaced.

For surveys between 2004 and 2013, the sampling within selected DSP, as described above, was conducted by the NCNCD centrally; since the 2015 survey, the sampling was performed by the provincial CDCs and approved by the NCNCD.

Sample size calculation
Sample size of each round of CCDRFS was calculated using N = $deff\,\frac{u^2 p(1-p)}{d^2}$
where u = 1.96 (corresponding to 95% confidence level), $deff$ is design efficiency (1.5 to 3.5), r is relative error (20%), and $d = r \times p$. p is the prevalence of factor studied for calculation. By using diabetes prevalence from previous national surveys (7-10%), the table below shows the calculated target sample sizes in each survey.

Survey	2004	2007	2010	2013	2015	2018
**Target sample size**	33,180	52,020	96,870	175,485	182,376	183,813

Sample weights
Across all CCDRFS surveys, we developed sample weights to account for multi-stage sampling design, post-stratification and, for the 2015 and 2018 surveys only, non-response within household as all members were selected. For an individual in the sample, his/her sample weights were developed as follows.

1. Base weights for multi-stage design (Wdesign)
   Wdesign = Wd1 × Wd2 × Wd3 × Wd4 × Wd5 × Wd6
   - Wd1 is the total number of counties (rural) or districts (urban) in the stratum divided by the number of selected DSPs (PSU) in the stratum where the individual was from;
   - Wd2 is the total number of townships (rural) or subdistricts (urban) in the PSU where the individual belonged divided by the number of selected townships or subdistricts;
   - Wd3 is the total number of villages (rural) or residential areas (urban) in the township or subdistrict where the individual was from divided by the number of selected villages or residential areas;
   - Wd4 is the total number of groups in the village or residential area where the individual was from;
   - Wd5 is the total number of households in the group where the individual belonged divided by the number of selected households in the group where the individual was from.
   - Wd6 is the total number of eligible adults in the household where the individual was from (2013 or earlier) or 1 (since 2015).

2. Non-response weights (Wnr) in the 2015 and 2018 surveys
   Wnr = the number of eligible adults in the household where the individual was from divided by the number of participating adults in the household.

3. Post-stratification weights (Wps)
   Stratifications included: province (31 levels), urban or rural (2 levels), gender (2 levels), age group (10 levels: 18-24, 25-29, 30-34, 35-39, 40-44, 45-49, 50-54, 55-59, 60-64, 65-69). The 2010 census population was also stratified in the same way. In k^{th} stratum, the post-stratification weights (Wps,k) are:

$$\text{Wps}, \text{k} = \frac{\text{Population in the } \text{k}^{\text{th}} \text{ stratum of the 2010 census population}}{\text{Sum of Wdesign} \times \text{Wnr for all individuals in the } \text{k}^{\text{th}} \text{ stratum}}$$

Appendix 2. Data collection of CCDRFS.

Overview

CCDRFS field work was coordinated by the NCNCD and implemented by the local CDCs. All filed work staff received mandatory training provided by certified instructors from the NCNCD and hosted by provincial CDCs.

The field work in each round of survey started in August, and in most rounds, finished in the same year. In 2013, 2015 and 2018, remaining visits were completed by June of the next year. Trained interviewers from local CDCs carried out the face-to-face interview, physical measurement, and biochemical sample collection and pre-treatment. Interview was conducted during a home visit. Physical measurement and biochemical sample collection were arranged at a community health centre.

Home visits

During home visit, the head of household or an adult who knew well the details of the household (e.g., economic and environmental information) was interviewed first using the household questionnaire that assessed the eligibility of all household members. Then, all eligible subjects were given a personal questionnaire that covered demographic characteristics, lifestyle factors, and history of chronic diseases. At the end of the home visit, the investigator gave each subject an appointment letter, instructing the location, time and precautions of the physical measurements.

Physical measurements

All subjects were invited to a community health centre to attend a physical measurement session. Subjects who did not complete their personal interviews during home visits also completed their interviewers in the service centre. Weight and height were measured using a standard protocol (Appendix Table 1).

Data management

From 2004 to 2013, all data were recorded on paper questionnaires first, and then entered into computer via a bespoke data management system, and delivered to the NCNCD via email or an online data transfer system. All the data entry and processing were done by the local CDCs, except in 2007 when the data entry was outsourced to a company. Since 2015, NCNCD deployed an integrated platform consisting of a tablet-assisted interview system and an online information management system, which was adopted by all local CDCs. The data of the questionnaire and physical measurement was collected electronically using the tablet-assisted system. The internet-based information management system was used to generate samples (as described in Appendix 1), on-site identity confirmation, quality control, physical examination reports, and data download.

Appendix 3. Analysis plan.

The aim of the analysis is to describe the trends from 2004 to 2018 in mean BMI and prevalence of obesity and overweight in China by subgroups of population, and examine how the trends may have changed before and after 2010.

Study participants
Men and women aged 18-69 years

Main outcomes
Mean BMI, prevalence of obesity (BMI ≥30 kg/m^2), prevalence of overweight (BMI ≥25 kg/m^2)

Secondary outcomes
Prevalence of obesity by Chinese cut-off points (BMI ≥28 kg/m^2), prevalence of overweight by Chinese cut-off points (BMI ≥24 kg/m^2)

Stratification
Sex (male, female), **age** (18-29, 30-39, 40-49, 50-59, 60-69 years), **locality of residence** (rural, urban), **education** (no education, primary, secondary, college), **region** (East, South, North, Central, Northeast, Southwest, Northwest), **occupation** (agriculture related, other manual work, non-manual work, not working, retired)

Interactions of grouping are limited to sex and place of residence plus one other group.

Statistics
Outcome (main and secondary) levels, overall and in groups as defined in 'Stratification'.

- SAS is used to account for clustering, stratification and sample weights in the sample design.
- Standard error and 95% confidence interval are estimated using *proc surveymeans* procedure in SAS, which uses Taylor series linearisation and accounts for finite population correction.[4]

Change in outcome (main and secondary) levels from 2004 to 2010 and from 2010 to 2018, overall and in groups as defined in 'Stratification'.

- Annual change in mean BMI (kg/m^2):

$$\text{annual change} = \frac{(\text{level}_{t2} - \text{level}_{t1})}{(t_2 - t_1)}$$

- Annual relative change in prevalence of obesity and overweight:

$$\text{annual relative change} = \left(\frac{\text{level}_{t2}}{\text{level}_{t1}}\right)^{\frac{1}{t_2 - t_1}} - 1$$

95% confidence interval for the annual change in mean BMI is calculated using the Welch's t-test. The statistical significance of the difference in the annual changes between 2004-2010 and 2010-2018 in mean BMI is also tested using the Welch's t-test.

95% confidence interval for the annual relative change in prevalence of obesity and overweight is calculated using 100,000 simulations, drawing from independent binomial distributions decided by the prevalence and effective sample sizes (calculated via standard errors) at the beginning and the end years. It is reported as the 2.5th and 97.5th percentiles from the simulation. Statistical significance of the difference in the annual relative changes between 2004-2010 and 2010-2018 is also tested using the same simulation method.

Missing data

Individuals with missing demographic information or height or weight measurements are excluded from the analyses.

References

1. Yang G, Hu J, Rao KQ, Ma J, Rao C, Lopez AD. Mortality registration and surveillance in China: History, current situation and challenges. *Popul Health Metr* 2005; **3**: 3.
2. Yang G. [Selection of DSP points in second stage and their presentation]. *Zhonghua Liu Xing Bing Xue Za Zhi* 1992; **13**: 197-201.
3. Zhou MG, Jiang Y, Huang ZJ, Wu F. Adjustment and representativeness evaluation of national disease surveillance points system. *Dis Surveill* 2010; **25**: 239-44.
4. SAS Institute Inc. SAS/STAT® 14.3 User's Guide The SURVEYMEANS Procedure. SAS/STAT® 143 User's Guide. Cary, NC: SAS Institute Inc.; 2017.

Appendix Figure 1. Trends in standardised mean height from 2004 to 2018, by sex and urban-rural locality.

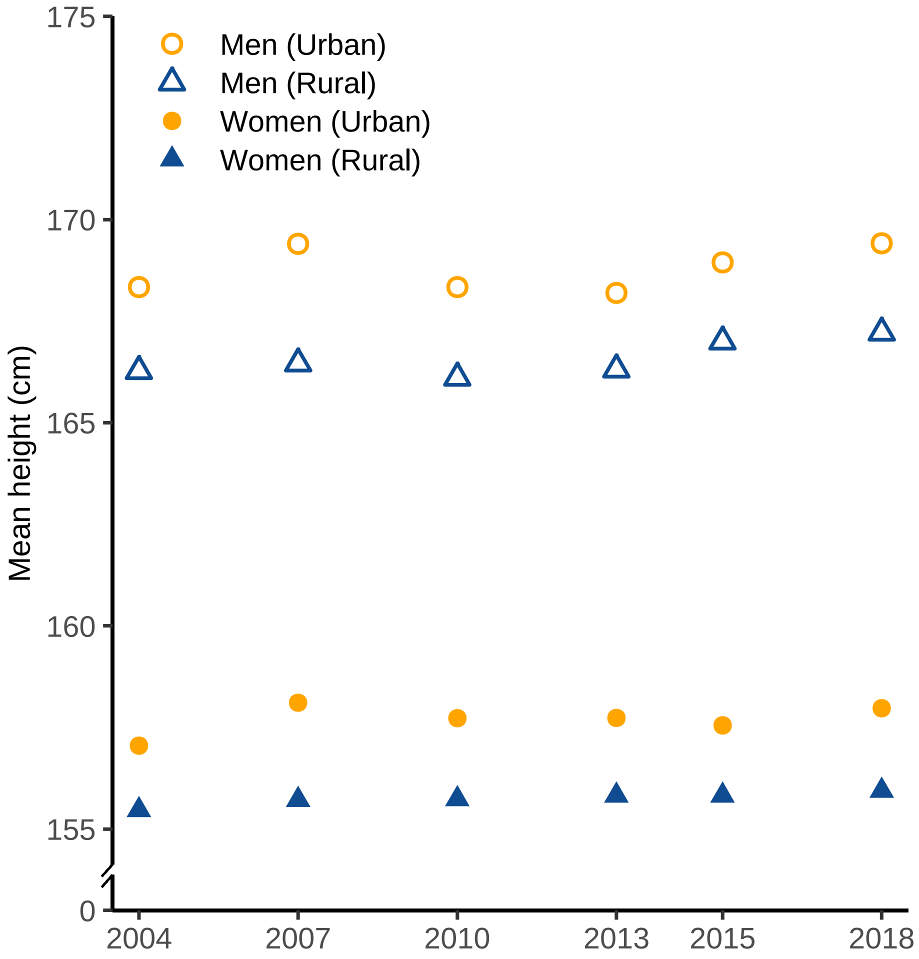

Appendix Figure 2. Change in BMI distribution from 2004 to 2018, by sex and urban-rural locality.

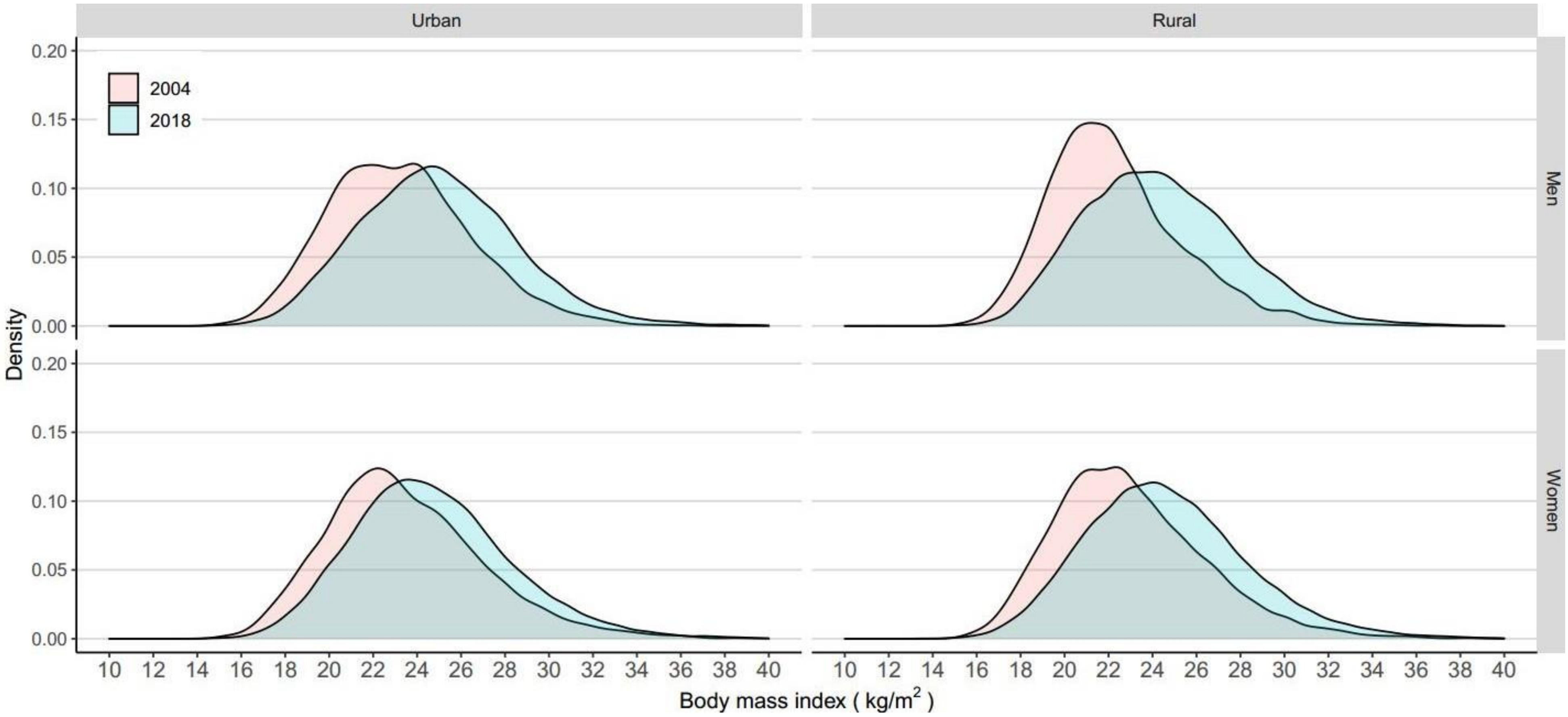

Appendix Figure 3. Trends in standard deviation of BMI from 2004 to 2018, by sex and urban-rural locality.

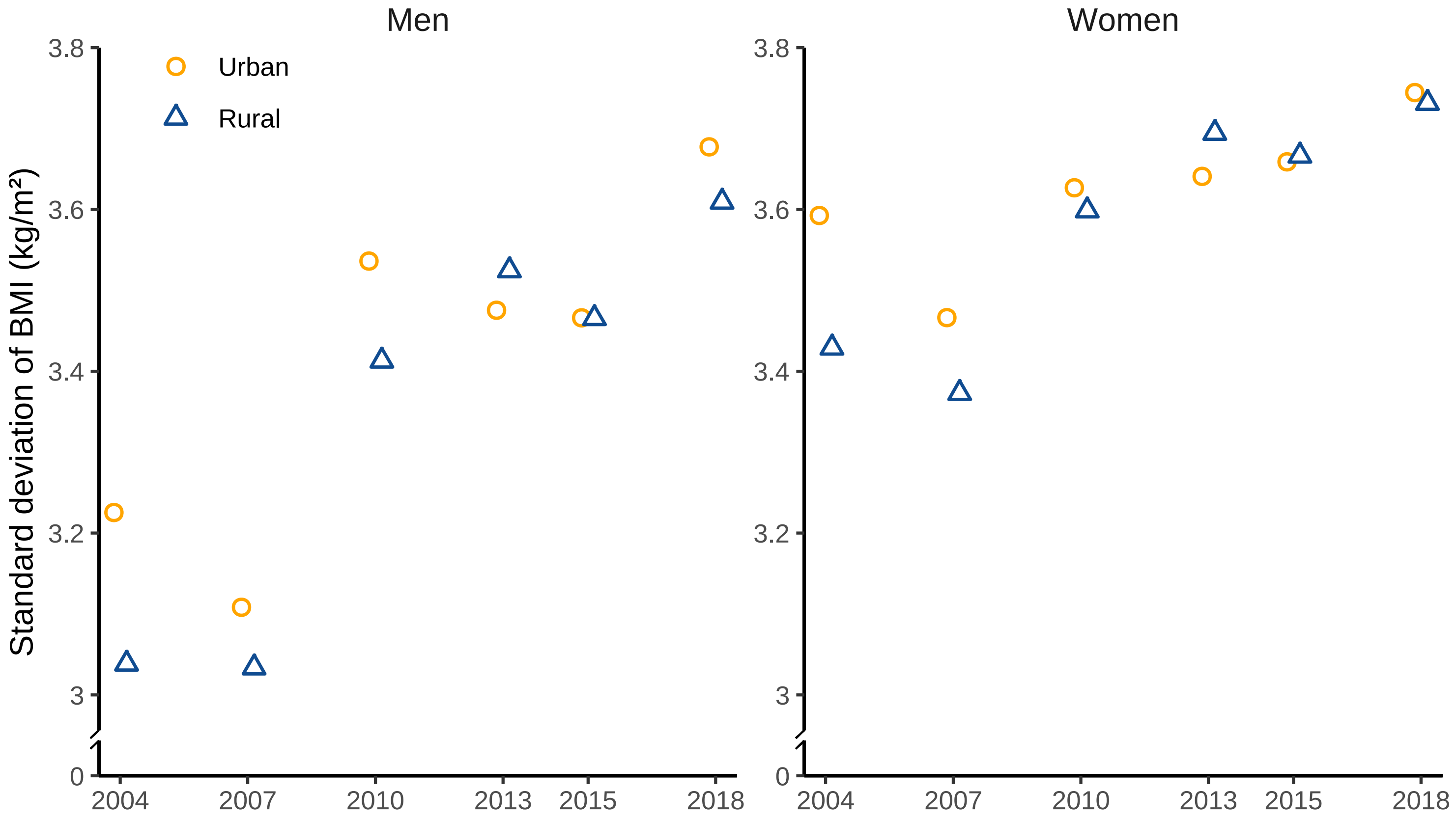

Appendix Figure 4. Trends in standardised prevalence of obesity from 2004 to 2018 in (A) urban and (B) rural areas, by sex and age group.

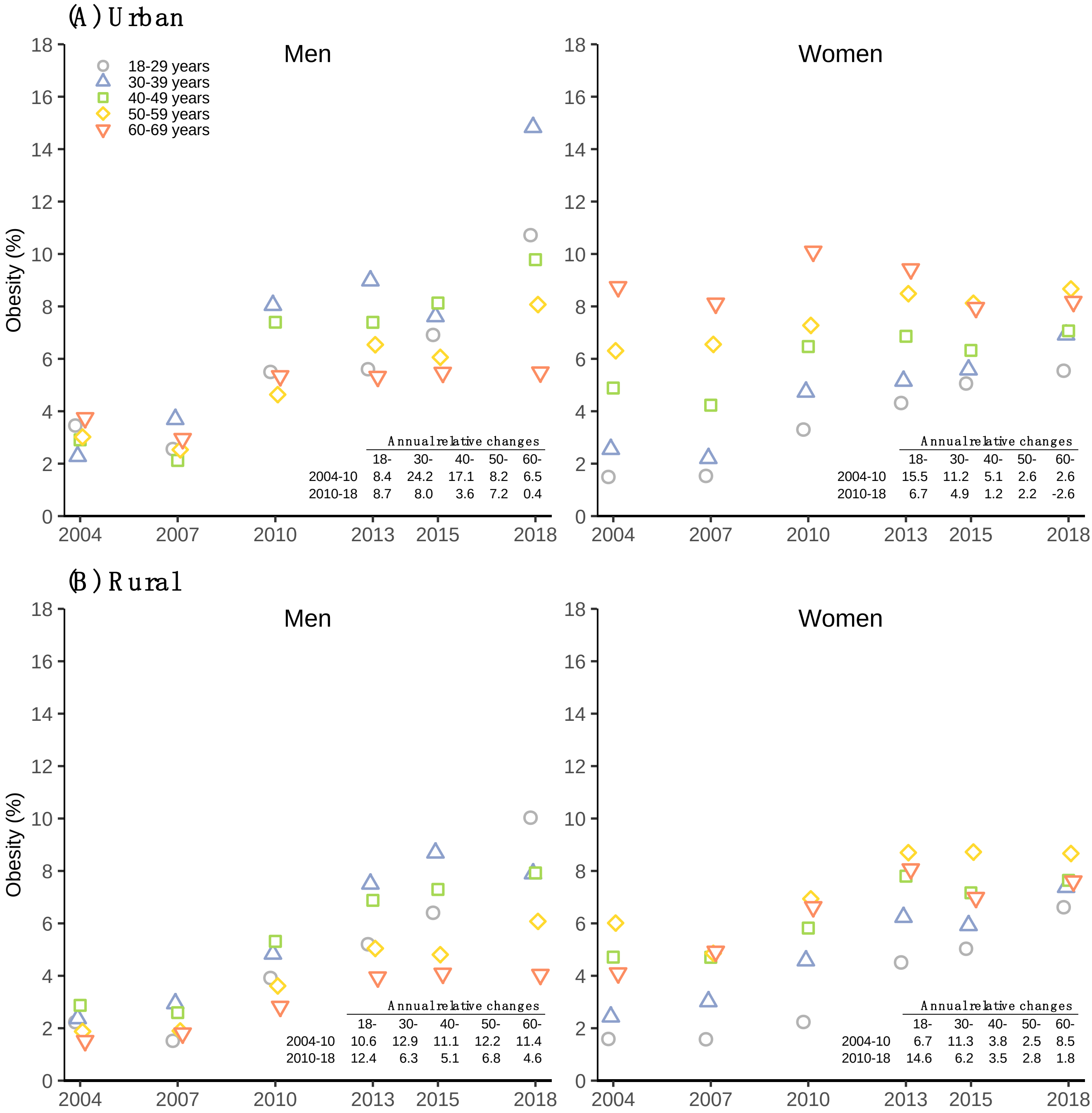

Appendix Figure 5. Trends in standardised prevalence of obesity from 2004 to 2018 in (A) urban and (B) rural areas, by sex and education.

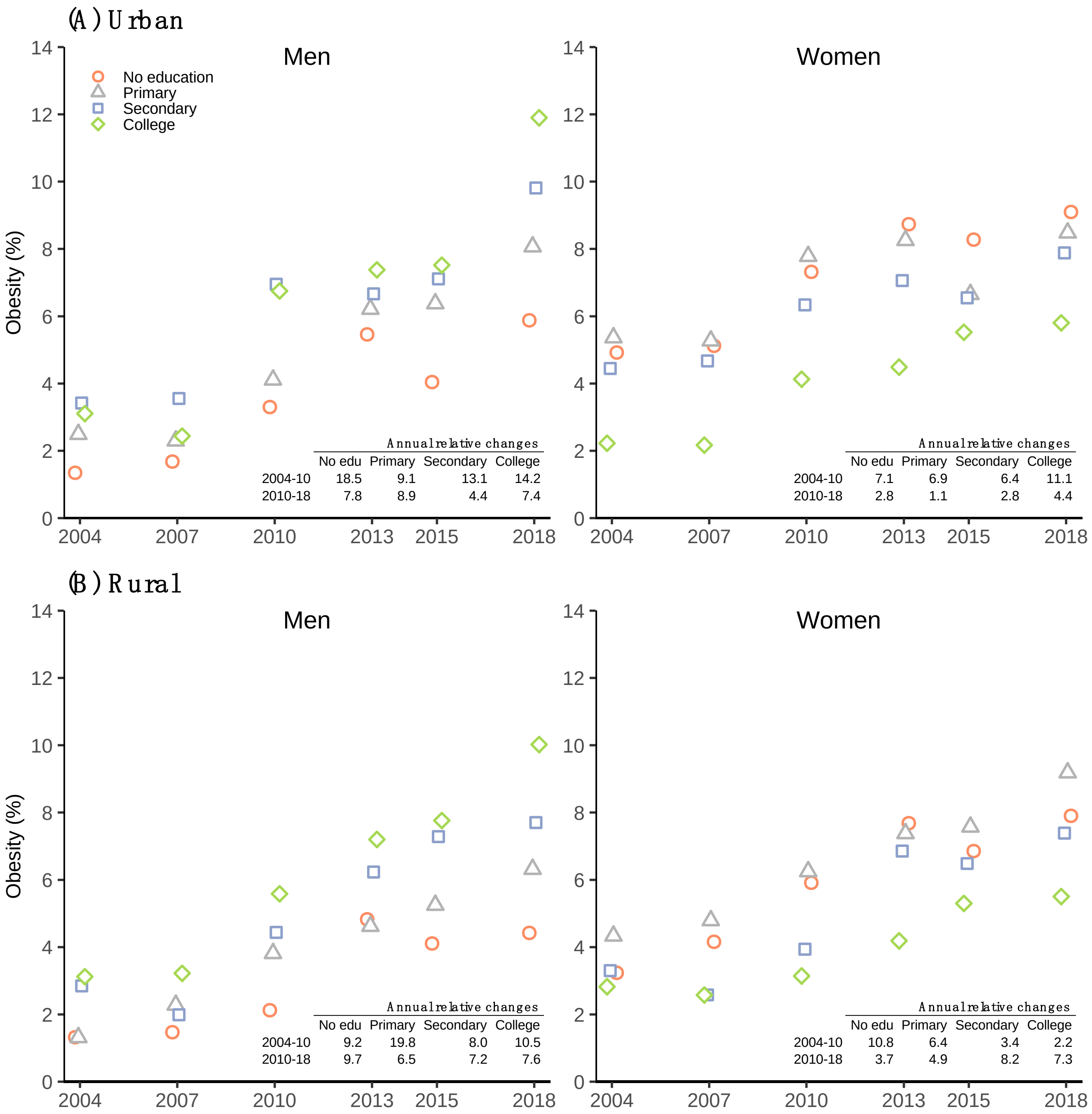

Appendix Figure 6. Trends in standardised mean BMI from 2004 to 2018 in (A) urban and (B) rural areas, by sex and occupation.

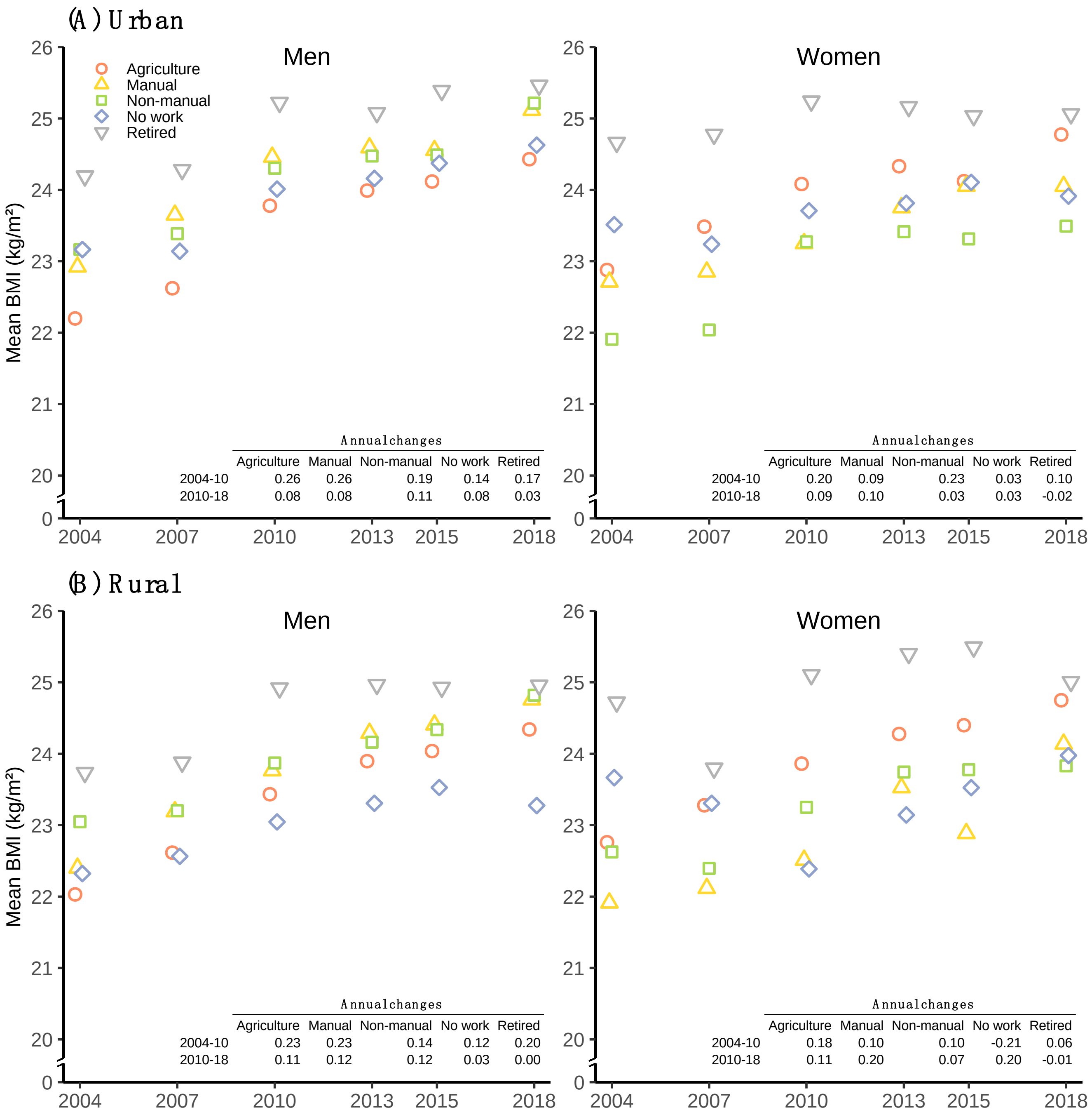

Appendix Figure 7. Trends in standardised prevalence of obesity from 2004 to 2018 in (A) urban and (B) rural areas, by sex and occupation.

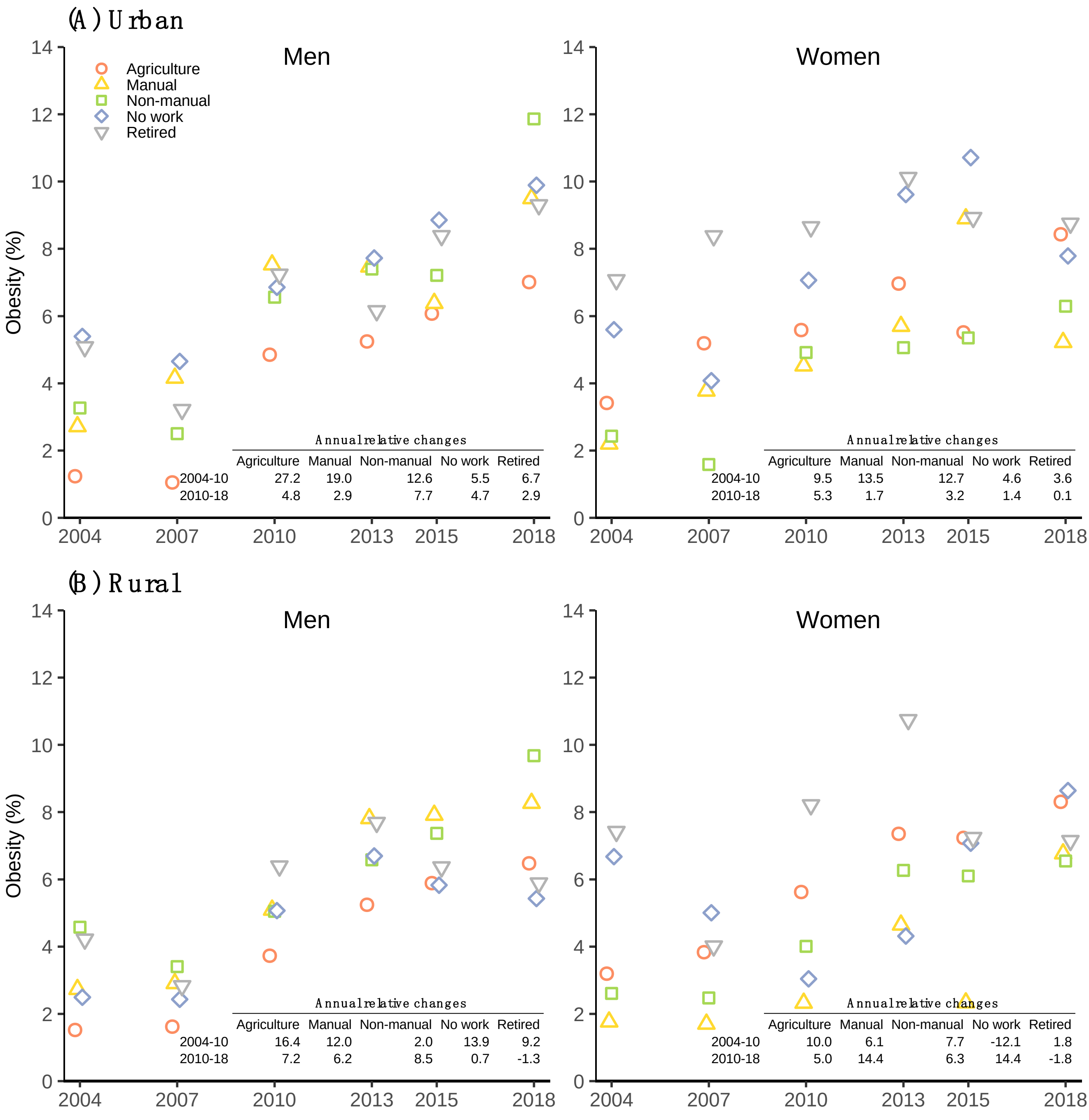

Appendix Figure 8. Trends in standardised mean BMI from 2004 to 2018 in (A) urban and (B) rural areas, by sex and region.

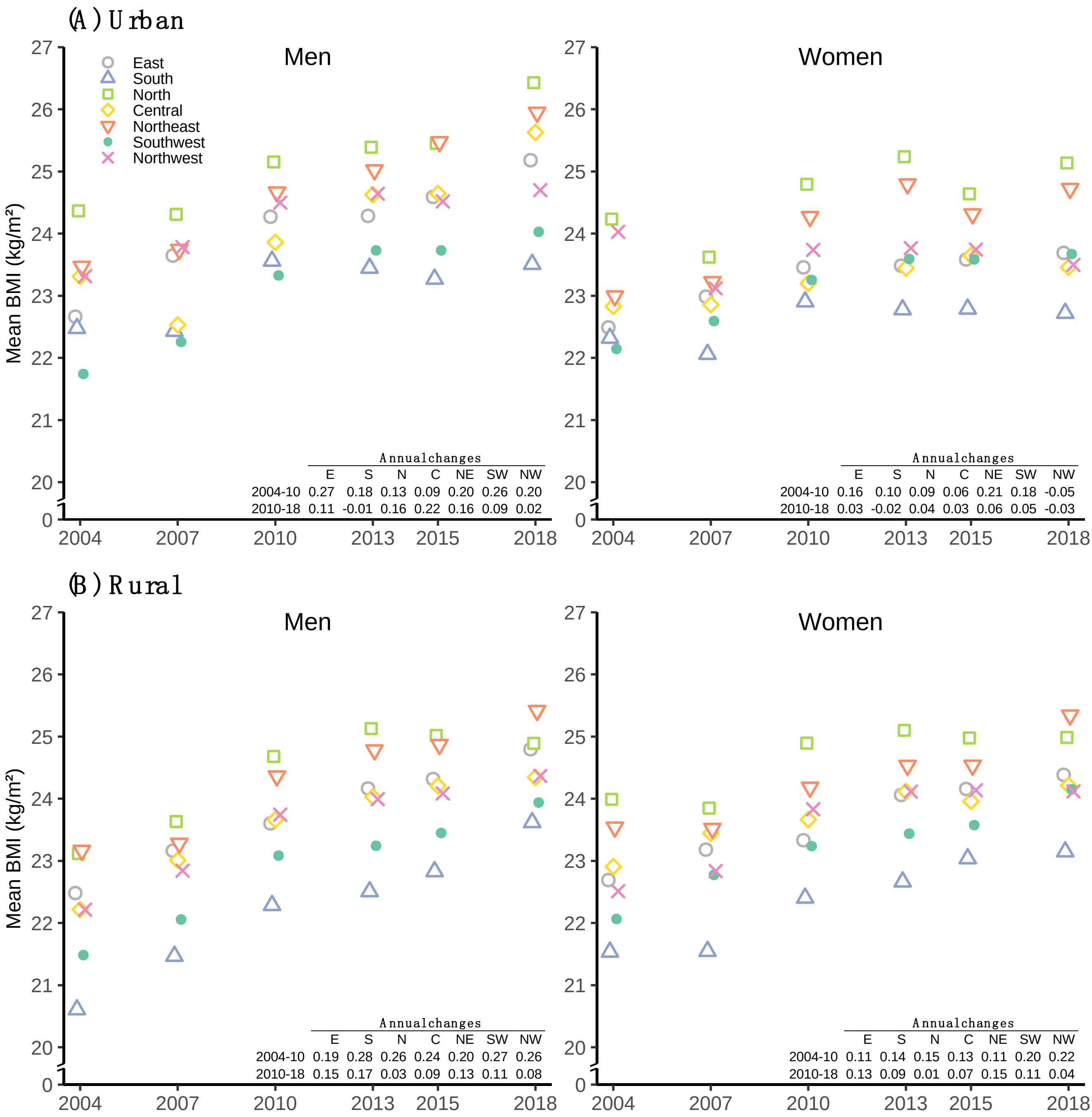

Appendix Figure 9. Trends in standardised prevalence of obesity from 2004 to 2018 in (A) urban and (B) rural areas, by sex and region.

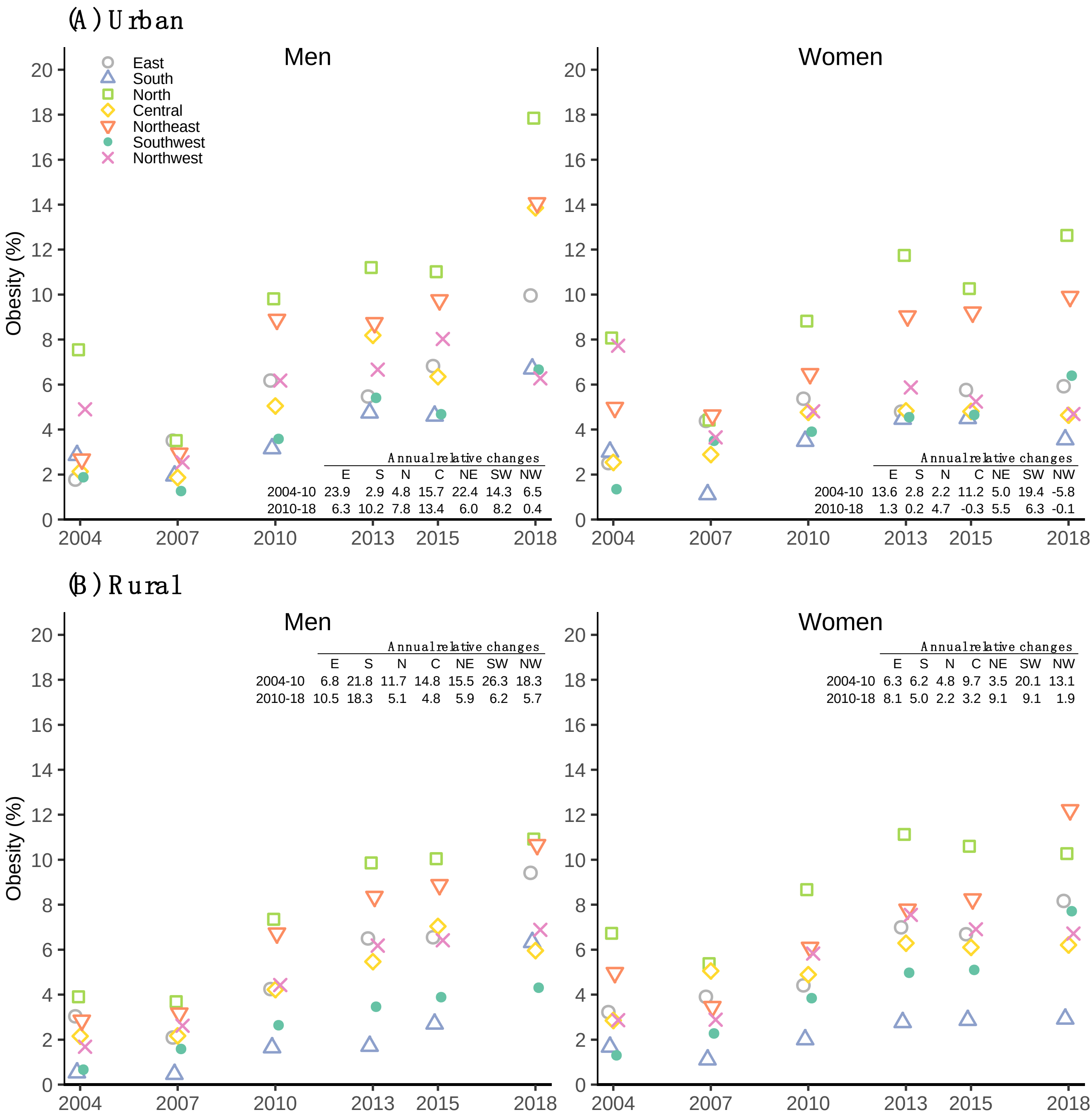

Appendix Figure 10. Trends in mean BMI and prevalence of obesity and overweight from 2004-2018, using data from DSPs that have been included in all survey rounds, by sex and urban-rural locality.

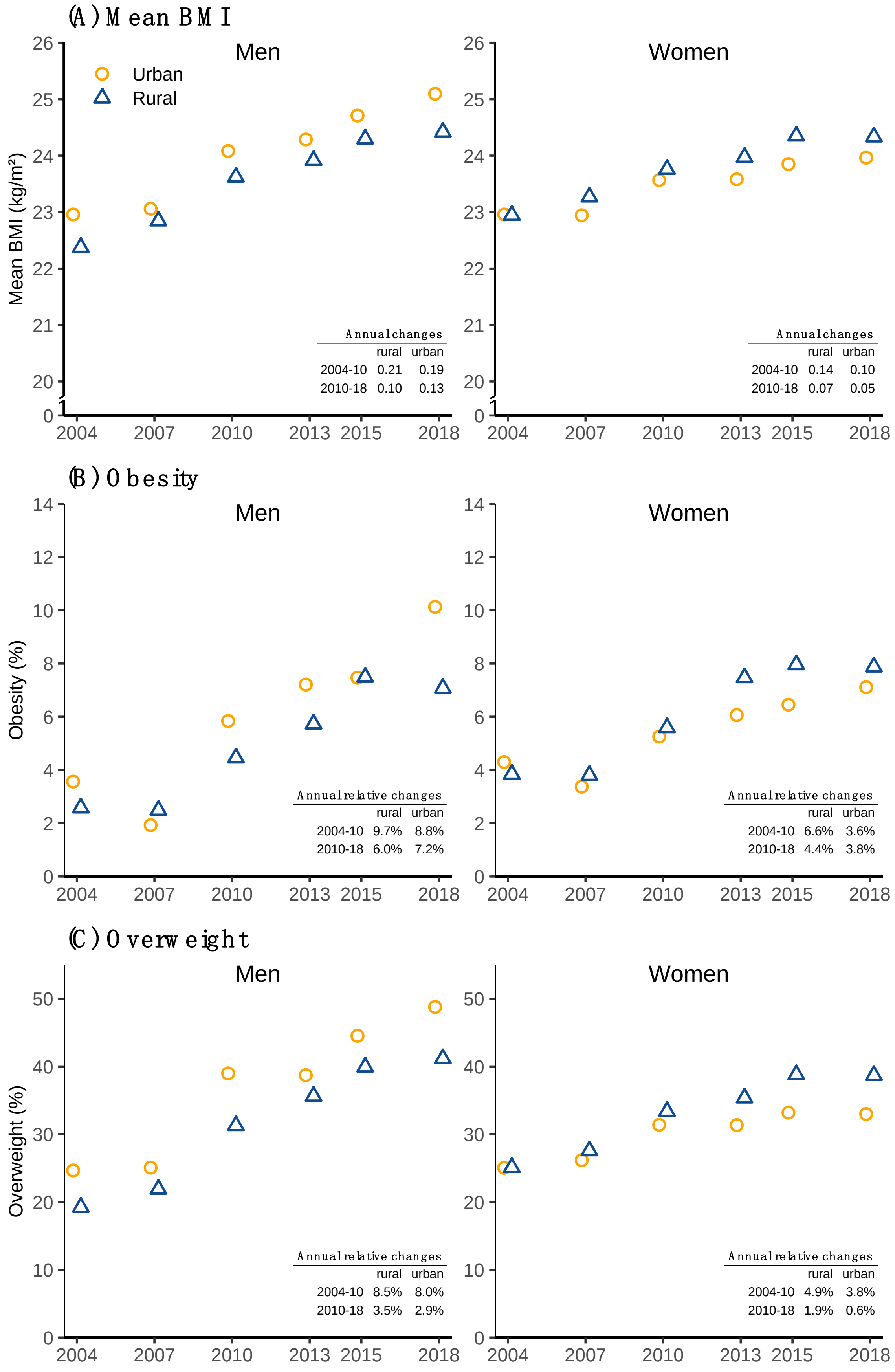

Appendix Table 1. Standardised mean BMI, prevalence of obesity and prevalence of overweight. See Appendix Table 3 for results using Chinese BMI cut-offs for obesity and overweight.

	2004	2007	2010	2013	2015	2018
**Men**						
**Mean (& 95%CI) BMI** (kg/m^2)						
Overall	22.5 (22.3, 22.8)	23.0 (22.8, 23.2)	23.8 (23.7, 24.0)	24.2 (24.0, 24.3)	24.3 (24.1, 24.5)	24.7 (24.6, 24.9)
Urban	23.0 (22.7, 23.2)	23.3 (23.0, 23.6)	24.2 (24.0, 24.5)	24.4 (24.2, 24.6)	24.5 (24.2, 24.8)	25.1 (24.8, 25.3)
Rural	22.3 (21.9, 22.6)	22.8 (22.6, 23.0)	23.6 (23.4, 23.8)	24.0 (23.8, 24.2)	24.2 (23.9, 24.4)	24.5 (24.3, 24.7)
**Prevalence (& 95%CI) of obesity** (%)						
Overall	2.6 (2.0, 3.1)	2.4 (2.0, 2.8)	5.1 (4.5, 5.6)	6.3 (5.7, 6.9)	6.8 (6.2, 7.4)	9.0 (8.3, 9.7)
Urban	3.0 (2.0, 4.0)	2.7 (2.1, 3.4)	6.4 (5.4, 7.3)	6.9 (6.0, 7.8)	7.1 (6.3, 7.9)	10.6 (9.4, 11.8)
Rural	2.3 (1.6, 3.0)	2.2 (1.8, 2.6)	4.3 (3.7, 4.9)	5.9 (5.2, 6.6)	6.5 (5.7, 7.4)	7.6 (6.7, 8.6)
**Prevalence (& 95%CI) of overweight** (%)						
Overall	20.9 (18.6, 23.2)	24.1 (22.1, 26.2)	34.5 (32.8, 36.2)	38.1 (36.6, 39.7)	39.8 (38.0, 41.6)	45.0 (43.3, 46.7)
Urban	25.0 (22.0, 28.1)	28.1 (24.8, 31.4)	39.4 (36.9, 42.0)	40.7 (38.6, 42.7)	42.0 (39.1, 44.8)	48.1 (45.3, 51.0)
Rural	18.5 (15.4, 21.5)	21.8 (19.3, 24.3)	31.5 (29.4, 33.6)	36.6 (34.5, 38.8)	38.2 (35.8, 40.5)	42.4 (40.3, 44.4)
**Women**						
**Mean (& 95%CI) BMI** (kg/m^2)						
Overall	22.9 (22.6, 23.1)	23.0 (22.8, 23.2)	23.6 (23.4, 23.8)	23.9 (23.8, 24.1)	23.9 (23.8, 24.1)	24.1 (24.0, 24.2)
Urban	22.9 (22.6, 23.2)	22.9 (22.7, 23.2)	23.7 (23.4, 23.9)	23.8 (23.6, 24.0)	23.7 (23.5, 23.9)	23.9 (23.6, 24.1)
Rural	22.9 (22.5, 23.2)	23.1 (22.8, 23.4)	23.6 (23.4, 23.8)	24.0 (23.8, 24.2)	24.1 (23.9, 24.2)	24.3 (24.1, 24.5)
**Prevalence (& 95%CI) of obesity** (%)						
Overall	3.7 (3.0, 4.4)	3.6 (3.1, 4.2)	5.2 (4.6, 5.7)	6.6 (6.1, 7.1)	6.5 (5.9, 7.0)	7.2 (6.7, 7.8)
Urban	3.9 (2.7, 5.1)	3.7 (2.9, 4.5)	5.6 (4.9, 6.3)	6.2 (5.5, 6.9)	6.2 (5.5, 6.9)	6.9 (6.1, 7.8)
Rural	3.6 (2.7, 4.5)	3.6 (2.8, 4.4)	4.9 (4.2, 5.6)	6.8 (6.1, 7.4)	6.6 (5.9, 7.4)	7.5 (6.7, 8.3)
**Prevalence (& 95%CI) of overweight** (%)						
Overall	24.6 (22.2, 27.1)	25.6 (23.5, 27.7)	31.5 (29.8, 33.3)	35.3 (33.7, 36.8)	34.4 (32.9, 35.9)	36.7 (35.2, 38.2)
Urban	25.1 (22.1, 28.2)	25.8 (23.3, 28.4)	31.7 (29.3, 34.2)	33.8 (31.4, 36.2)	31.8 (29.9, 33.6)	34.4 (32.1, 36.8)
Rural	24.4 (20.9, 27.8)	25.5 (22.5, 28.4)	31.4 (29.1, 33.7)	36.1 (34.2, 38.0)	36.3 (34.3, 38.2)	38.5 (36.6, 40.3)

Appendix Table 1. Continued.

**Both**						
**Mean (& 95%CI) BMI** (kg/m^2)						
Overall	22.7 (22.5, 22.9)	23.0 (22.8, 23.2)	23.7 (23.6, 23.9)	24.0 (23.9, 24.2)	24.1 (24.0, 24.2)	24.4 (24.3, 24.6)
Urban	22.9 (22.6, 23.2)	23.1 (22.9, 23.4)	24.0 (23.7, 24.2)	24.1 (23.9, 24.3)	24.1 (23.9, 24.3)	24.5 (24.3, 24.7)
Rural	22.6 (22.2, 22.9)	22.9 (22.7, 23.2)	23.6 (23.4, 23.8)	24.0 (23.8, 24.2)	24.1 (23.9, 24.3)	24.4 (24.2, 24.5)
**Prevalence (& 95%CI) of obesity** (%)						
Overall	3.1 (2.5, 3.7)	3.0 (2.6, 3.4)	5.1 (4.6, 5.6)	6.4 (6.0, 6.9)	6.6 (6.1, 7.2)	8.1 (7.6, 8.7)
Urban	3.5 (2.5, 4.5)	3.2 (2.6, 3.9)	6.0 (5.2, 6.8)	6.6 (5.8, 7.3)	6.7 (6.0, 7.4)	8.8 (7.9, 9.7)
Rural	2.9 (2.2, 3.6)	2.9 (2.4, 3.4)	4.6 (4.0, 5.2)	6.4 (5.8, 6.9)	6.6 (5.8, 7.4)	7.6 (6.9, 8.3)
**Prevalence (& 95%CI) of overweight** (%)						
Overall	22.7 (20.5, 25.0)	24.9 (23.0, 26.7)	33.0 (31.5, 34.6)	36.7 (35.3, 38.1)	37.1 (35.6, 38.6)	40.9 (39.5, 42.2)
Urban	25.1 (22.2, 27.9)	27.0 (24.4, 29.5)	35.7 (33.4, 38.0)	37.3 (35.2, 39.4)	37.0 (34.8, 39.1)	41.4 (39.1, 43.6)
Rural	21.4 (18.2, 24.5)	23.6 (21.1, 26.1)	31.5 (29.5, 33.5)	36.4 (34.4, 38.3)	37.2 (35.2, 39.2)	40.4 (38.8, 42.0)

Appendix Table 2. Annual change in standardised mean BMI, prevalence of obesity and prevalence of overweight from 2004 to 2010 and from 2010 to 2018 in different age groups, education levels, occupation groups and regions, by sex.

**Change (& 95%CI) in mean BMI** (kg/m^2)		**Men (overall)**			**Women (overall)**		
		**2004-2010**	**2010-2018**	**p***	**2004-2010**	**2010-2018**	**p***
Age group	18-29 years	0.21 (0.14, 0.28)	0.13 (0.08, 0.18)	0.0442	0.12 (0.07, 0.17)	0.09 (0.05, 0.13)	0.2341
	30-39 years	0.22 (0.18, 0.27)	0.13 (0.09, 0.16)	0.0037	0.12 (0.07, 0.17)	0.05 (0.02, 0.08)	0.0282
	40-49 years	0.24 (0.18, 0.29)	0.10 (0.07, 0.13)	0.0001	0.12 (0.07, 0.18)	0.04 (0.01, 0.07)	0.0118
	50-59 years	0.18 (0.13, 0.24)	0.12 (0.09, 0.14)	0.0367	0.12 (0.06, 0.18)	0.06 (0.03, 0.09)	0.0670
	60-69 years	0.21 (0.15, 0.27)	0.08 (0.05, 0.11)	0.0005	0.16 (0.09, 0.24)	0.04 (0.01, 0.08)	0.0074
Education	No education	0.23 (0.17, 0.28)	0.09 (0.05, 0.13)	0.0003	0.18 (0.12, 0.24)	0.09 (0.06, 0.12)	0.0172
	Primary	0.23 (0.18, 0.28)	0.12 (0.09, 0.15)	0.0020	0.16 (0.09, 0.23)	0.10 (0.06, 0.13)	0.0885
	Secondary	0.21 (0.15, 0.28)	0.11 (0.07, 0.14)	0.0055	0.11 (0.06, 0.17)	0.09 (0.06, 0.13)	0.3065
	College	0.18 (0.12, 0.23)	0.11 (0.07, 0.15)	0.0429	0.11 (0.05, 0.16)	0.03 (0.00, 0.07)	0.0457
Region	East	0.22 (0.14, 0.30)	0.14 (0.09, 0.19)	0.0787	0.13 (0.04, 0.22)	0.08 (0.03, 0.13)	0.2043
	South	0.20 (0.04, 0.37)	0.11 (0.03, 0.18)	0.1720	0.10 (-0.01, 0.22)	0.05 (-0.01, 0.10)	0.2060
	North	0.22 (0.13, 0.31)	0.10 (0.03, 0.17)	0.0249	0.13 (0.03, 0.22)	0.03 (-0.03, 0.08)	0.0559
	Central	0.20 (0.08, 0.33)	0.12 (0.05, 0.18)	0.1546	0.11 (-0.01, 0.22)	0.06 (0.00, 0.12)	0.2645
	Northeast	0.20 (0.15, 0.26)	0.15 (0.10, 0.20)	0.1210	0.14 (0.06, 0.21)	0.10 (0.05, 0.14)	0.2180
	Southwest	0.27 (0.21, 0.32)	0.10 (0.06, 0.15)	0.0001	0.19 (0.11, 0.27)	0.09 (0.04, 0.14)	0.0448
	Northwest	0.24 (0.12, 0.35)	0.07 (0.01, 0.13)	0.0109	0.13 (0.00, 0.27)	0.00 (-0.06, 0.06)	0.0574
Occupation	Agriculture related	0.24 (0.18, 0.30)	0.11 (0.08, 0.14)	0.0007	0.19 (0.12, 0.25)	0.11 (0.08, 0.14)	0.0314
	Other manual work	0.23 (0.16, 0.31)	0.11 (0.05, 0.16)	0.0092	0.10 (-0.02, 0.22)	0.16 (0.09, 0.23)	0.2422
	Non-manual work	0.16 (0.11, 0.22)	0.12 (0.08, 0.15)	0.1102	0.18 (0.13, 0.23)	0.05 (0.02, 0.08)	0.0002
	Not working	0.12 (0.02, 0.23)	0.05 (-0.03, 0.14)	0.1868	-0.06 (-0.17, 0.04)	0.09 (0.03, 0.16)	0.0194
	Retired	0.18 (0.11, 0.26)	0.03 (-0.03, 0.09)	0.0015	0.09 (0.02, 0.16)	-0.02 (-0.08, 0.03)	0.0246

* p-value for difference in annual changes for 2004-2010 versus 2010-2018.

Appendix Table 2. Continued.

% change (& 95%CI) in obesity prevalence		Men (overall) 2004-2010	Men (overall) 2010-2018	Men (overall) p*	Women (overall) 2004-2010	Women (overall) 2010-2018	Women (overall) p*
Age group	18-29 years	9.2 (2.6, 17.0)	10.8 (7.2, 14.4)	0.3490	9.9 (1.7, 20.2)	10.9 (6.7, 15.2)	0.4123
	30-39 years	17.5 (11.6, 24.3)	8.1 (5.7, 10.6)	0.0042	11.1 (6.1, 16.7)	5.6 (3.2, 8.1)	0.0461
	40-49 years	13.3 (8.1, 19.2)	4.7 (2.6, 6.8)	0.0035	4.1 (-0.3, 9.1)	2.6 (0.5, 4.7)	0.3055
	50-59 years	10.1 (3.2, 18.4)	7.1 (4.7, 9.6)	0.2541	2.5 (-1.1, 6.4)	2.6 (0.9, 4.4)	0.4727
	60-69 years	8.7 (2.4, 16.0)	3.1 (0.4, 5.9)	0.0881	5.6 (0.9, 10.9)	0.1 (-1.8, 2.2)	0.0365
Education	No education	10.8 (1.3, 23.4)	9.2 (5.5, 13.3)	0.4208	9.3 (4.6, 14.4)	3.6 (1.7, 5.7)	0.0294
	Primary	16.0 (9.7, 23.2)	7.3 (4.2, 10.5)	0.0190	6.3 (1.7, 11.5)	4.0 (1.4, 6.5)	0.2324
	Secondary	9.8 (4.1, 16.4)	6.2 (4.0, 8.5)	0.1680	4.4 (-0.5, 10.1)	5.9 (3.6, 8.3)	0.3140
	College	12.5 (7.2, 18.5)	7.6 (5.2, 10.1)	0.0781	7.7 (2.2, 13.8)	5.4 (2.3, 8.5)	0.2704
Region	East	13.6 (4.7, 24.8)	8.5 (5.1, 12.0)	0.1900	9.2 (2.1, 17.8)	4.9 (2.0, 7.9)	0.1809
	South	6.3 (-7.5, 27.1)	15.0 (8.0, 22.9)	0.1823	1.6 (-7.7, 13.6)	3.1 (-1.7, 8.3)	0.3931
	North	8.9 (2.5, 16.8)	6.9 (4.2, 9.5)	0.3299	3.6 (-2.8, 11.1)	3.6 (0.6, 6.7)	0.4905
	Central	14.8 (-0.2, 38.2)	7.7 (2.5, 13.5)	0.2604	10.0 (2.2, 18.5)	2.4 (-1.8, 7.0)	0.0881
	Northeast	18.2 (12.5, 24.3)	6.3 (3.3, 9.5)	0.0015	3.9 (-0.3, 8.4)	7.3 (4.3, 10.2)	0.1367
	Southwest	20.1 (9.3, 35.4)	7.4 (3.6, 11.1)	0.0190	19.8 (13.9, 26.4)	8.1 (4.7, 11.5)	0.0020
	Northwest	12.0 (-0.5, 30.7)	3.5 (-1.9, 9.0)	0.1606	4.7 (-6.3, 20.0)	0.6 (-4.1, 5.6)	0.3066
Occupation	Agriculture related	18.1 (11.3, 26.0)	6.7 (4.2, 9.4)	0.0030	9.7 (4.7, 15.3)	5.1 (3.0, 7.2)	0.0736
	Other manual work	14.9 (7.6, 23.7)	4.6 (1.0, 8.2)	0.0124	9.8 (0.4, 21.5)	8.3 (2.5, 14.0)	0.4282
	Non-manual work	7.4 (2.5, 13.0)	8.2 (5.9, 10.5)	0.3971	10.2 (5.5, 15.4)	4.6 (2.4, 6.9)	0.0343
	Not working	6.8 (-3.8, 22.1)	3.3 (-2.1, 8.7)	0.3354	-1.5 (-10.0, 8.0)	5.2 (-0.7, 11.5)	0.1562
	Retired	7.0 (-0.3, 16.2)	2.6 (-3.6, 8.1)	0.2123	3.2 (-0.9, 7.5)	0.0 (-2.5, 2.5)	0.1279

* p-value for difference in annual changes for 2004-2010 versus 2010-2018.

Appendix Table 2. Continued.

% change (& 95%CI) in overweight prevalence		Men (overall)			Women (overall)		
		**2004-2010**	**2010-2018**	**p***	**2004-2010**	**2010-2018**	**p***
Age group	18-29 years	10.4 (7.0, 14.3)	4.4 (2.9, 5.9)	0.0018	5.9 (2.2, 10.0)	4.5 (2.6, 6.3)	0.2804
	30-39 years	8.6 (6.3, 10.9)	3.5 (2.5, 4.5)	0.0002	5.0 (2.9, 7.3)	2.0 (0.9, 3.2)	0.0193
	40-49 years	8.8 (6.6, 11.2)	2.8 (1.9, 3.7)	<0.0001	3.7 (1.9, 5.6)	1.1 (0.3, 2.0)	0.0131
	50-59 years	7.4 (4.8, 10.3)	3.3 (2.4, 4.2)	0.0035	3.2 (1.4, 5.0)	1.4 (0.5, 2.3)	0.0699
	60-69 years	8.1 (5.1, 11.4)	2.6 (1.6, 3.7)	0.0008	4.3 (1.8, 6.9)	0.9 (0.0, 1.9)	0.0152
Education	No education	13.6 (9.7, 18.1)	4.4 (2.9, 5.9)	<0.0001	6.2 (3.7, 8.9)	2.5 (1.6, 3.5)	0.0092
	Primary	11.4 (8.7, 14.4)	4.1 (3.0, 5.3)	<0.0001	5.4 (2.9, 8.1)	2.7 (1.6, 3.7)	0.0382
	Secondary	7.7 (5.2, 10.3)	3.2 (2.4, 4.2)	0.0009	3.9 (1.6, 6.3)	2.8 (1.8, 3.9)	0.2375
	College	6.7 (4.7, 8.8)	2.6 (1.7, 3.6)	0.0009	3.1 (0.4, 5.9)	1.3 (-0.3, 2.9)	0.1684
Region	East	8.4 (4.7, 12.5)	3.6 (2.4, 5.0)	0.0154	4.8 (0.9, 9.2)	2.6 (1.0, 4.3)	0.1915
	South	9.8 (1.5, 20.2)	4.4 (1.4, 7.8)	0.1626	2.1 (-2.6, 7.5)	3.3 (1.0, 5.5)	0.3436
	North	7.0 (3.3, 11.3)	2.6 (0.9, 4.2)	0.0215	3.7 (0.9, 6.7)	0.1 (-1.5, 1.6)	0.0261
	Central	8.6 (2.9, 15.3)	4.0 (2.0, 6.1)	0.0949	4.4 (0.0, 9.4)	1.8 (-0.2, 3.9)	0.1870
	Northeast	7.5 (4.8, 10.4)	3.9 (2.6, 5.3)	0.0250	4.0 (1.4, 6.8)	2.4 (1.2, 3.7)	0.1799
	Southwest	17.4 (13.1, 22.2)	3.6 (1.8, 5.4)	<0.0001	10.1 (5.5, 15.1)	3.2 (1.3, 5.3)	0.0107
	Northwest	10.3 (4.4, 17.4)	2.4 (0.5, 4.3)	0.0091	4.3 (-0.7, 10.2)	0.7 (-1.2, 2.6)	0.1270
Occupation	Agriculture related	11.8 (8.6, 15.2)	4.1 (3.0, 5.2)	<0.0001	6.8 (4.2, 9.6)	3.0 (2.0, 4.0)	0.0082
	Other manual work	8.2 (5.3, 11.3)	2.7 (1.2, 4.2)	0.0016	5.3 (-0.5, 12.0)	4.9 (2.1, 8.0)	0.4643
	Non-manual work	5.7 (3.8, 7.6)	2.8 (2.0, 3.7)	0.0075	6.3 (4.0, 8.7)	1.6 (0.5, 2.7)	0.0008
	Not working	5.0 (1.3, 9.0)	2.7 (0.4, 4.9)	0.1836	-3.2 (-6.3, 0.0)	4.1 (1.9, 6.4)	0.0012
	Retired	5.4 (3.0, 8.1)	0.7 (-0.6, 1.9)	0.0006	2.2 (0.2, 4.1)	-0.6 (-1.9, 0.7)	0.0277

* p-value for difference in annual changes for 2004-2010 versus 2010-2018.

Appendix Table 3. Annual change in standardised mean BMI, prevalence of obesity and prevalence of overweight from 2004 to 2010 and from 2010 to 2018 in different age groups, education levels, occupation groups and regions, by sex and urban or rural residence.

**Change (& 95%CI) in mean BMI** (kg/m²)		**Urban men**			**Rural men**		
		**2004-2010**	**2010-2018**	**p***	**2004-2010**	**2010-2018**	**p***
Age group	18-29 years	0.21 (0.11, 0.31)	0.12 (0.04, 0.20)	0.1285	0.21 (0.12, 0.31)	0.12 (0.06, 0.18)	0.0666
	30-39 years	0.23 (0.17, 0.28)	0.13 (0.07, 0.18)	0.0246	0.22 (0.16, 0.29)	0.10 (0.06, 0.15)	0.0054
	40-49 years	0.24 (0.17, 0.31)	0.08 (0.04, 0.12)	0.0003	0.24 (0.16, 0.31)	0.10 (0.06, 0.14)	0.0034
	50-59 years	0.19 (0.10, 0.27)	0.08 (0.04, 0.12)	0.0272	0.18 (0.11, 0.25)	0.13 (0.10, 0.17)	0.1446
	60-69 years	0.21 (0.11, 0.31)	0.03 (-0.02, 0.08)	0.0045	0.21 (0.14, 0.28)	0.09 (0.05, 0.12)	0.0048
Education	No education	0.26 (0.17, 0.34)	0.08 (0.00, 0.17)	0.0089	0.22 (0.16, 0.28)	0.09 (0.05, 0.14)	0.0022
	Primary	0.25 (0.17, 0.32)	0.09 (0.02, 0.15)	0.0037	0.22 (0.16, 0.29)	0.13 (0.09, 0.17)	0.0159
	Secondary	0.21 (0.13, 0.28)	0.09 (0.04, 0.14)	0.0178	0.22 (0.13, 0.30)	0.11 (0.07, 0.15)	0.0246
	College	0.19 (0.12, 0.26)	0.11 (0.05, 0.16)	0.0483	0.16 (0.08, 0.25)	0.10 (0.04, 0.16)	0.1570
Region	East	0.27 (0.18, 0.36)	0.11 (0.05, 0.18)	0.0154	0.19 (0.05, 0.32)	0.15 (0.09, 0.21)	0.3279
	South	0.18 (0.07, 0.29)	-0.01 (-0.08, 0.06)	0.0044	0.28 (0.15, 0.41)	0.17 (0.08, 0.26)	0.1248
	North	0.13 (0.06, 0.20)	0.16 (0.09, 0.23)	0.3247	0.26 (0.16, 0.37)	0.03 (-0.05, 0.10)	0.0007
	Central	0.09 (-0.01, 0.19)	0.22 (0.12, 0.32)	0.0513	0.24 (0.09, 0.40)	0.09 (0.01, 0.16)	0.0687
	Northeast	0.20 (0.12, 0.28)	0.16 (0.09, 0.23)	0.2785	0.20 (0.13, 0.27)	0.13 (0.07, 0.20)	0.1203
	Southwest	0.26 (0.17, 0.36)	0.09 (0.01, 0.17)	0.0137	0.27 (0.20, 0.34)	0.11 (0.05, 0.16)	0.0019
	Northwest	0.20 (0.00, 0.39)	0.02 (-0.08, 0.13)	0.0856	0.26 (0.13, 0.38)	0.08 (0.01, 0.15)	0.0142
Occupation	Agriculture related	0.26 (0.18, 0.35)	0.08 (0.02, 0.14)	0.0031	0.23 (0.16, 0.30)	0.11 (0.08, 0.15)	0.0054
	Other manual work	0.26 (0.15, 0.36)	0.08 (-0.01, 0.17)	0.0146	0.23 (0.13, 0.33)	0.12 (0.06, 0.19)	0.0664
	Non-manual work	0.19 (0.13, 0.25)	0.11 (0.06, 0.16)	0.0458	0.14 (0.05, 0.23)	0.12 (0.07, 0.17)	0.3766
	Not working	0.14 (0.00, 0.28)	0.08 (-0.04, 0.19)	0.2712	0.12 (-0.05, 0.29)	0.03 (-0.08, 0.13)	0.2116
	Retired	0.17 (0.09, 0.25)	0.03 (-0.04, 0.10)	0.0071	0.20 (0.05, 0.35)	0.00 (-0.08, 0.09)	0.0252

* p-value for difference in annual changes for 2004-2010 versus 2010-2018.

Appendix Table 2. Continued.

Change (& 95%CI) in mean BMI (kg/m^2)		Urban women 2004-2010	Urban women 2010-2018	Urban women p*	Rural women 2004-2010	Rural women 2010-2018	Rural women p*
Age group	18-29 years	0.16 (0.09, 0.23)	0.06 (0.00, 0.12)	0.0428	0.09 (0.03, 0.16)	0.12 (0.07, 0.17)	0.2752
	30-39 years	0.15 (0.10, 0.20)	0.02 (-0.02, 0.07)	0.0013	0.10 (0.03, 0.17)	0.08 (0.03, 0.12)	0.3059
	40-49 years	0.11 (0.04, 0.19)	0.00 (-0.04, 0.05)	0.0203	0.12 (0.05, 0.20)	0.07 (0.03, 0.10)	0.1026
	50-59 years	0.09 (0.01, 0.17)	0.02 (-0.03, 0.06)	0.1160	0.14 (0.06, 0.22)	0.08 (0.04, 0.12)	0.1565
	60-69 years	0.12 (0.03, 0.21)	-0.03 (-0.08, 0.01)	0.0047	0.18 (0.09, 0.27)	0.07 (0.02, 0.12)	0.0400
Education	No education	0.14 (0.05, 0.22)	0.06 (0.01, 0.12)	0.1358	0.20 (0.12, 0.28)	0.10 (0.06, 0.14)	0.0206
	Primary	0.21 (0.09, 0.33)	0.04 (-0.02, 0.09)	0.0106	0.15 (0.06, 0.24)	0.11 (0.06, 0.16)	0.2657
	Secondary	0.15 (0.06, 0.23)	0.07 (0.02, 0.11)	0.0828	0.10 (0.02, 0.17)	0.11 (0.06, 0.15)	0.4308
	College	0.16 (0.09, 0.23)	0.02 (-0.03, 0.07)	0.0039	0.01 (-0.09, 0.11)	0.06 (0.01, 0.12)	0.2155
Region	East	0.16 (0.07, 0.25)	0.03 (-0.03, 0.09)	0.0274	0.11 (-0.04, 0.25)	0.13 (0.06, 0.20)	0.3946
	South	0.10 (0.02, 0.17)	-0.02 (-0.10, 0.05)	0.0463	0.14 (-0.04, 0.33)	0.09 (0.04, 0.15)	0.3142
	North	0.09 (-0.09, 0.28)	0.04 (-0.04, 0.12)	0.3251	0.15 (0.04, 0.26)	0.01 (-0.06, 0.08)	0.0393
	Central	0.06 (-0.03, 0.16)	0.03 (-0.06, 0.12)	0.3580	0.13 (-0.03, 0.28)	0.07 (-0.01, 0.15)	0.2870
	Northeast	0.21 (0.08, 0.35)	0.06 (0.00, 0.11)	0.0224	0.11 (0.02, 0.19)	0.15 (0.09, 0.21)	0.2733
	Southwest	0.18 (0.11, 0.26)	0.05 (-0.01, 0.12)	0.0234	0.20 (0.09, 0.30)	0.11 (0.04, 0.18)	0.1561
	Northwest	-0.05 (-0.24, 0.14)	-0.03 (-0.12, 0.06)	0.4428	0.22 (0.08, 0.36)	0.04 (-0.03, 0.11)	0.0225
Occupation	Agriculture related	0.20 (0.10, 0.30)	0.09 (0.03, 0.15)	0.0432	0.18 (0.11, 0.26)	0.11 (0.07, 0.15)	0.0669
	Other manual work	0.09 (0.00, 0.18)	0.10 (0.02, 0.18)	0.4447	0.10 (-0.08, 0.28)	0.20 (0.10, 0.30)	0.1941
	Non-manual work	0.23 (0.16, 0.29)	0.03 (-0.02, 0.07)	<0.0001	0.10 (0.03, 0.17)	0.07 (0.03, 0.12)	0.2593
	Not working	0.03 (-0.11, 0.18)	0.03 (-0.06, 0.11)	0.4735	-0.21 (-0.36, -0.07)	0.20 (0.11, 0.29)	0.0001
	Retired	0.10 (0.01, 0.18)	-0.02 (-0.08, 0.04)	0.0333	0.06 (-0.05, 0.18)	-0.01 (-0.11, 0.08)	0.2038

* p-value for difference in annual changes for 2004-2010 versus 2010-2018.

Appendix Table 2. Continued.

% change (& 95%CI) in obesity prevalence		Urban men			Rural men		
		**2004-2010**	**2010-2018**	**p***	**2004-2010**	**2010-2018**	**p***
Age group	18-29 years	8.4 (0.2, 17.9)	8.7 (4.2, 13.5)	0.4687	10.6 (0.4, 24.7)	12.4 (6.8, 17.9)	0.3760
	30-39 years	24.2 (14.4, 37.1)	8.0 (4.9, 11.1)	0.0012	12.9 (5.9, 21.4)	6.3 (3.2, 9.5)	0.0727
	40-49 years	17.1 (10.3, 25.2)	3.6 (0.7, 6.5)	0.0006	11.1 (4.1, 19.3)	5.1 (2.2, 8.2)	0.0969
	50-59 years	8.2 (-1.2, 21.6)	7.2 (4.0, 10.5)	0.4627	12.2 (2.8, 24.5)	6.8 (3.6, 10.2)	0.1881
	60-69 years	6.5 (-1.9, 16.4)	0.4 (-3.4, 4.7)	0.1485	11.4 (2.5, 22.6)	4.6 (1.0, 8.5)	0.1224
Education	No education	18.5 (0.7, 51.2)	7.8 (1.4, 15.4)	0.1996	9.2 (-2.0, 24.9)	9.7 (5.1, 14.7)	0.4484
	Primary	9.1 (-1.1, 21.4)	8.9 (2.6, 15.5)	0.4879	19.8 (11.8, 29.3)	6.5 (2.9, 10.3)	0.0044
	Secondary	13.1 (4.7, 23.9)	4.4 (1.5, 7.5)	0.0434	8.0 (0.6, 17.1)	7.2 (4.1, 10.5)	0.4455
	College	14.2 (6.9, 23.1)	7.4 (4.5, 10.3)	0.0662	10.5 (3.1, 19.3)	7.6 (2.9, 12.2)	0.2913
Region	East	23.9 (12.3, 38.7)	6.3 (1.9, 11.2)	0.0089	6.8 (-3.7, 21.9)	10.5 (5.8, 15.2)	0.2795
	South	2.9 (-11.4, 23.6)	10.2 (2.5, 19.2)	0.2349	21.8 (4.1, 44.2)	18.3 (5.0, 31.7)	0.3997
	North	4.8 (-1.6, 12.8)	7.8 (5.0, 10.5)	0.2306	11.7 (3.0, 23.3)	5.1 (1.7, 8.6)	0.1196
	Central	15.7 (8.6, 23.8)	13.4 (9.0, 17.8)	0.3334	14.8 (-5.1, 44.8)	4.8 (-1.6, 12.9)	0.2479
	Northeast	22.4 (14.1, 31.7)	6.0 (2.0, 10.2)	0.0021	15.5 (8.7, 23.2)	5.9 (2.0, 9.9)	0.0226
	Southwest	14.3 (-3.7, 44.2)	8.2 (1.4, 15.8)	0.3502	26.3 (18.0, 36.7)	6.2 (2.1, 10.2)	<0.0001
	Northwest	6.5 (-12.9, 34.6)	0.4 (-9.9, 11.8)	0.3550	18.3 (7.0, 32.9)	5.7 (0.2, 11.4)	0.0470
Occupation	Agriculture related	27.2 (12.9, 48.7)	4.8 (0.4, 9.5)	0.0031	16.4 (9.0, 25.3)	7.2 (4.2, 10.4)	0.0256
	Other manual work	19.0 (9.9, 30.6)	2.9 (-1.8, 7.5)	0.0025	12.0 (1.4, 27.2)	6.2 (1.0, 11.3)	0.2214
	Non-manual work	12.6 (6.1, 20.0)	7.7 (5.0, 10.6)	0.1235	2.0 (-5.0, 10.5)	8.5 (4.6, 12.5)	0.0979
	Not working	5.5 (-7.4, 25.5)	4.7 (-2.1, 11.6)	0.4999	13.9 (-0.1, 34.4)	0.7 (-7.9, 9.0)	0.0936
	Retired	6.7 (-1.5, 17.5)	2.9 (-4.0, 8.9)	0.2825	9.2 (-6.7, 38.1)	-1.3 (-11.4, 7.7)	0.1835

* p-value for difference in annual changes for 2004-2010 versus 2010-2018.

Appendix Table 2. Continued.

% change (& 95%CI) in obesity prevalence		Urban women 2004-2010	Urban women 2010-2018	p*	Rural women 2004-2010	Rural women 2010-2018	p*
Age group	18-29 years	15.5 (2.9, 34.0)	6.7 (1.0, 12.4)	0.1551	6.7 (-3.8, 20.9)	14.6 (8.6, 20.6)	0.1453
	30-39 years	11.2 (4.1, 19.7)	4.9 (1.5, 8.4)	0.0896	11.3 (4.6, 19.1)	6.2 (2.9, 9.6)	0.1308
	40-49 years	5.1 (-1.9, 13.7)	1.2 (-2.1, 4.6)	0.2066	3.8 (-1.9, 10.1)	3.5 (0.8, 6.3)	0.4762
	50-59 years	2.6 (-2.7, 8.7)	2.2 (-0.4, 4.9)	0.4721	2.5 (-2.2, 7.9)	2.8 (0.6, 5.2)	0.4552
	60-69 years	2.6 (-3.0, 9.1)	-2.6 (-5.1, 0.1)	0.0837	8.5 (1.8, 16.3)	1.8 (-1.0, 4.8)	0.0603
Education	No education	7.1 (0.0, 15.5)	2.8 (-0.5, 6.3)	0.1860	10.8 (4.9, 17.6)	3.7 (1.4, 6.2)	0.0295
	Primary	6.9 (-1.6, 17.7)	1.1 (-2.3, 4.8)	0.1534	6.4 (1.0, 12.6)	4.9 (1.7, 8.2)	0.3507
	Secondary	6.4 (0.0, 14.2)	2.8 (-0.3, 5.8)	0.1969	3.4 (-3.6, 12.2)	8.2 (4.9, 11.5)	0.1518
	College	11.1 (4.4, 18.8)	4.4 (0.7, 8.1)	0.0706	2.2 (-6.7, 12.9)	7.3 (2.3, 12.7)	0.2093
Region	East	13.6 (7.8, 19.7)	1.3 (-2.1, 4.9)	0.0026	6.3 (-4.5, 21.4)	8.1 (3.7, 12.7)	0.3744
	South	2.8 (-5.6, 12.7)	0.2 (-6.5, 6.6)	0.3432	6.2 (-12.1, 29.7)	5.0 (-0.2, 11.1)	0.4870
	North	2.2 (-7.3, 15.2)	4.7 (0.6, 9.0)	0.3333	4.8 (-3.7, 15.1)	2.2 (-1.9, 6.6)	0.3383
	Central	11.2 (3.0, 20.0)	-0.3 (-6.1, 5.3)	0.0336	9.7 (-0.4, 21.0)	3.2 (-1.9, 9.2)	0.1873
	Northeast	5.0 (-2.8, 15.3)	5.5 (1.9, 8.9)	0.4447	3.5 (-1.5, 8.9)	9.1 (4.6, 13.5)	0.0877
	Southwest	19.4 (13.5, 25.5)	6.3 (1.4, 11.2)	0.0031	20.1 (12.0, 29.5)	9.1 (4.9, 13.5)	0.0244
	Northwest	-5.8 (-19.7, 20.0)	-0.1 (-7.0, 7.4)	0.2588	13.1 (2.3, 26.4)	1.9 (-4.0, 8.3)	0.0790
Occupation	Agriculture related	9.5 (-0.3, 23.1)	5.3 (1.7, 9.0)	0.2710	10.0 (4.5, 16.4)	5.0 (2.7, 7.5)	0.0863
	Other manual work	13.5 (3.7, 26.6)	1.7 (-5.0, 7.7)	0.0372	6.1 (-8.2, 27.0)	14.4 (5.6, 23.7)	0.2103
	Non-manual work	12.7 (6.4, 19.9)	3.2 (0.3, 6.2)	0.0103	7.7 (0.9, 15.9)	6.3 (3.0, 9.8)	0.3950
	Not working	4.6 (-7.1, 19.7)	1.4 (-5.5, 9.0)	0.3613	-12.1 (-24.5, 1.4)	14.4 (4.2, 26.8)	0.0050
	Retired	3.6 (-1.1, 8.9)	0.1 (-2.5, 2.8)	0.1419	1.8 (-6.7, 10.9)	-1.8 (-9.7, 5.7)	0.2947

* p-value for difference in annual changes for 2004-2010 versus 2010-2018.

Appendix Table 2. Continued.

% change (& 95%CI) in overweight prevalence		Urban men 2004-2010	Urban men 2010-2018	Urban men p*	Rural men 2004-2010	Rural men 2010-2018	Rural men p*
Age group	18-29 years	10.7 (5.7, 16.5)	3.5 (1.4, 5.7)	0.0082	10.3 (5.7, 15.7)	4.8 (2.7, 7.0)	0.0259
	30-39 years	6.8 (4.3, 9.4)	3.1 (1.8, 4.5)	0.0161	10.1 (6.8, 13.8)	3.4 (2.1, 4.7)	0.0003
	40-49 years	7.6 (5.2, 10.0)	1.8 (0.7, 2.9)	0.0001	9.8 (6.5, 13.4)	3.4 (2.2, 4.6)	0.0005
	50-59 years	7.5 (4.2, 11.0)	2.2 (1.1, 3.3)	0.0028	7.5 (3.7, 11.8)	3.9 (2.7, 5.1)	0.0540
	60-69 years	7.1 (3.4, 11.2)	1.3 (-0.3, 2.9)	0.0084	8.8 (4.7, 13.6)	3.1 (1.8, 4.4)	0.0079
Education	No education	12.3 (7.6, 17.5)	3.4 (0.8, 6.0)	0.0027	14.7 (9.6, 20.7)	4.7 (2.9, 6.5)	0.0003
	Primary	11.2 (7.3, 15.4)	2.3 (0.3, 4.3)	0.0002	11.7 (8.2, 15.6)	4.7 (3.4, 6.1)	0.0004
	Secondary	5.9 (3.1, 9.0)	2.7 (1.5, 3.9)	0.0357	8.9 (5.6, 12.7)	3.4 (2.2, 4.6)	0.0029
	College	7.7 (5.3, 10.2)	2.2 (1.0, 3.4)	0.0002	5.4 (2.1, 9.1)	3.1 (1.5, 4.7)	0.1417
Region	East	10.2 (6.4, 14.3)	2.5 (0.8, 4.3)	0.0010	7.2 (1.2, 14.6)	4.6 (2.8, 6.3)	0.2436
	South	7.8 (4.6, 11.0)	0.1 (-2.5, 2.6)	0.0013	17.6 (7.3, 30.6)	7.4 (3.2, 12.3)	0.0723
	North	3.3 (0.5, 6.3)	3.3 (1.7, 5.0)	0.4878	9.4 (4.5, 15.2)	1.3 (-1.0, 3.4)	0.0018
	Central	2.5 (-2.0, 7.7)	5.7 (3.3, 8.0)	0.1367	11.5 (4.4, 20.1)	3.3 (0.8, 6.0)	0.0338
	Northeast	8.4 (4.7, 12.6)	3.8 (2.2, 5.5)	0.0288	6.8 (3.3, 10.7)	3.7 (1.7, 5.7)	0.0986
	Southwest	12.8 (8.4, 17.4)	2.8 (0.4, 5.3)	0.0007	20.1 (15.1, 25.6)	3.9 (1.5, 6.5)	<0.0001
	Northwest	8.7 (0.0, 20.8)	0.4 (-2.5, 3.4)	0.0590	11.8 (5.0, 20.4)	3.2 (1.0, 5.3)	0.0097
Occupation	Agriculture related	11.8 (7.0, 17.2)	3.3 (1.1, 5.6)	0.0032	11.8 (8.1, 15.9)	4.2 (3.0, 5.5)	0.0002
	Other manual work	8.0 (4.5, 12.0)	1.7 (-0.6, 3.9)	0.0042	9.4 (5.0, 14.5)	3.7 (1.7, 5.6)	0.0187
	Non-manual work	6.7 (4.7, 8.8)	2.4 (1.3, 3.6)	0.0008	4.4 (1.3, 7.9)	3.2 (1.8, 4.6)	0.2687
	Not working	6.0 (1.3, 11.3)	3.2 (0.5, 6.0)	0.2016	4.1 (-1.7, 10.4)	1.9 (-1.8, 5.7)	0.3097
	Retired	5.7 (3.1, 8.6)	0.6 (-0.8, 1.9)	0.0006	4.6 (-0.6, 10.8)	0.7 (-1.9, 3.3)	0.1278

* p-value for difference in annual changes for 2004-2010 versus 2010-2018.

Appendix Table 2. Continued.

% change (&95%CI) in overweight prevalence		Urban women 2004-2010	Urban women 2010-2018	Urban women p*	Rural women 2004-2010	Rural women 2010-2018	Rural women p*
Age group	18-29 years	6.5 (1.4, 12.4)	4.5 (1.7, 7.4)	0.2875	5.7 (0.7, 11.5)	4.6 (2.2, 7.1)	0.3887
	30-39 years	6.3 (3.7, 8.9)	1.3 (-0.3, 3.0)	0.0041	4.4 (1.4, 7.6)	2.5 (1.0, 4.1)	0.1857
	40-49 years	3.4 (0.8, 6.1)	0.0 (-1.4, 1.5)	0.0343	3.9 (1.5, 6.5)	1.9 (0.9, 2.9)	0.0932
	50-59 years	2.5 (0.2, 4.8)	0.1 (-1.1, 1.3)	0.0700	3.6 (1.1, 6.3)	2.2 (1.0, 3.4)	0.2024
	60-69 years	2.5 (0.3, 4.8)	-0.6 (-1.6, 0.5)	0.0160	5.5 (1.8, 9.7)	1.6 (0.3, 3.1)	0.0474
Education	No education	4.4 (1.8, 7.1)	1.8 (0.2, 3.4)	0.0906	7.0 (3.7, 10.7)	2.7 (1.6, 3.9)	0.0154
	Primary	6.2 (2.7, 10.1)	0.9 (-0.5, 2.4)	0.0074	5.3 (2.2, 8.7)	3.2 (1.9, 4.5)	0.1507
	Secondary	4.0 (0.8, 7.5)	2.5 (1.1, 4.0)	0.2415	4.0 (0.8, 7.5)	3.0 (1.6, 4.4)	0.3126
	College	5.6 (2.3, 9.1)	0.7 (-1.2, 2.6)	0.0172	-1.1 (-5.1, 3.2)	2.4 (0.0, 4.9)	0.1130
Region	East	5.3 (2.3, 8.3)	1.7 (-0.3, 3.7)	0.0572	4.6 (-1.7, 12.3)	3.6 (1.1, 6.1)	0.4132
	South	1.1 (-2.1, 4.2)	0.1 (-2.6, 3.0)	0.3532	5.7 (-3.2, 18.3)	5.4 (2.9, 8.0)	0.4762
	North	2.4 (-1.8, 7.2)	0.4 (-2.0, 2.7)	0.2441	4.5 (0.8, 8.6)	-0.3 (-2.2, 1.6)	0.0244
	Central	2.3 (-1.9, 6.3)	0.9 (-2.8, 4.6)	0.3406	5.1 (-0.6, 12.0)	2.0 (-0.1, 4.3)	0.2050
	Northeast	8.5 (2.3, 16.5)	1.5 (-0.3, 3.2)	0.0216	2.5 (0.0, 5.1)	3.3 (1.9, 4.9)	0.3229
	Southwest	7.9 (4.9, 10.9)	2.2 (0.4, 4.1)	0.0061	11.2 (4.8, 18.6)	3.8 (1.1, 6.8)	0.0438
	Northwest	-2.1 (-7.1, 3.3)	-0.5 (-3.3, 2.6)	0.3394	8.5 (2.6, 15.6)	1.7 (-0.5, 4.1)	0.0342
Occupation	Agriculture related	7.9 (3.7, 12.7)	2.4 (0.5, 4.3)	0.0186	6.7 (3.6, 9.9)	3.1 (2.0, 4.3)	0.0277
	Other manual work	5.4 (-0.6, 11.9)	3.2 (-0.4, 7.1)	0.3081	5.0 (-4.4, 16.4)	6.7 (2.2, 11.9)	0.3926
	Non-manual work	7.6 (4.3, 11.1)	1.3 (-0.3, 3.0)	0.0024	4.7 (1.5, 8.1)	2.0 (0.5, 3.5)	0.0991
	Not working	0.4 (-3.5, 4.7)	1.7 (-1.0, 4.4)	0.3341	-9.7 (-14.9, -4.4)	9.3 (5.2, 13.9)	<0.0001
	Retired	2.3 (0.2, 4.5)	-0.7 (-2.2, 0.7)	0.0303	1.3 (-2.5, 5.2)	0.1 (-2.9, 3.1)	0.3423

* p-value for difference in annual changes for 2004-2010 versus 2010-2018.

Appendix Table 3. Standardised prevalence of obesity and overweight defined by Chinese BMI cut-offs (obesity: BMI ≥28 and overweight: ≥24 kg/m^2).

	2004	2007	2010	2013	2015	2018
**Men**						
**Prevalence (& 95%CI) of obesity** (%)						
Overall	6.1 (5.1, 7.0)	6.8 (5.9, 7.7)	12.3 (11.3, 13.3)	14.4 (13.5, 15.3)	15.1 (14.0, 16.3)	18.8 (17.6, 20.0)
Urban	7.2 (5.7, 8.7)	8.0 (6.5, 9.6)	14.6 (13.1, 16.2)	15.4 (14.1, 16.7)	16.0 (14.3, 17.7)	21.4 (19.3, 23.5)
Rural	5.4 (4.2, 6.6)	6.0 (5.1, 7.0)	10.9 (9.7, 12.1)	13.8 (12.7, 15.0)	14.5 (12.9, 16.1)	16.7 (15.3, 18.0)
**Prevalence (& 95%CI) of overweight** (%)						
Overall	29.1 (26.3, 31.9)	34.5 (32.1, 36.9)	44.3 (42.3, 46.2)	48.6 (47.0, 50.1)	50.7 (48.8, 52.6)	55.2 (53.4, 56.9)
Urban	35.7 (32.0, 39.4)	39.7 (36.0, 43.3)	49.9 (47.2, 52.6)	51.4 (49.3, 53.5)	52.9 (49.7, 56.1)	58.6 (55.6, 61.5)
Rural	25.2 (21.6, 28.7)	31.4 (28.5, 34.3)	40.9 (38.6, 43.3)	46.9 (44.8, 49.0)	49.1 (46.7, 51.6)	52.3 (50.2, 54.5)
**Women**						
**Prevalence (& 95%CI) of obesity** (%)						
Overall	8.2 (6.9, 9.6)	8.9 (7.8, 9.9)	11.7 (10.8, 12.7)	14.0 (13.2, 14.9)	13.3 (12.4, 14.2)	14.8 (13.9, 15.8)
Urban	8.6 (6.5, 10.6)	8.9 (7.5, 10.2)	12.4 (11.1, 13.8)	13.3 (12.0, 14.6)	12.4 (11.1, 13.7)	13.9 (12.4, 15.3)
Rural	8.0 (6.2, 9.8)	8.9 (7.4, 10.3)	11.3 (10.1, 12.5)	14.4 (13.3, 15.6)	14.0 (12.8, 15.2)	15.6 (14.3, 16.9)
**Prevalence (& 95%CI) of overweight** (%)						
Overall	33.1 (30.3, 35.9)	34.7 (32.3, 37.1)	41.0 (39.2, 42.9)	45.1 (43.4, 46.8)	44.7 (43.0, 46.4)	47.1 (45.4, 48.8)
Urban	33.5 (30.3, 36.8)	35.0 (32.2, 37.8)	41.6 (39.0, 44.2)	43.6 (40.9, 46.3)	41.8 (39.4, 44.1)	43.9 (41.3, 46.5)
Rural	32.8 (28.8, 36.8)	34.5 (31.1, 37.9)	40.7 (38.2, 43.2)	46.0 (43.9, 48.1)	46.8 (44.6, 49.0)	49.8 (47.7, 51.8)
**Both**						
**Prevalence (& 95%CI) of obesity** (%)						
Overall	7.1 (6.0, 8.2)	7.8 (7.0, 8.7)	12.0 (11.1, 12.9)	14.2 (13.4, 15.0)	14.2 (13.3, 15.2)	16.8 (15.9, 17.8)
Urban	7.9 (6.2, 9.5)	8.4 (7.1, 9.8)	13.6 (12.2, 14.9)	14.4 (13.2, 15.6)	14.2 (12.8, 15.6)	17.7 (16.1, 19.2)
Rural	6.7 (5.2, 8.1)	7.4 (6.4, 8.5)	11.1 (10.0, 12.2)	14.1 (13.1, 15.2)	14.3 (12.9, 15.6)	16.1 (15.0, 17.3)
**Prevalence (& 95%CI) of overweight** (%)						
Overall	31.1 (28.4, 33.7)	34.6 (32.4, 36.8)	42.7 (40.9, 44.4)	46.9 (45.4, 48.4)	47.7 (46.1, 49.4)	51.2 (49.7, 52.7)
Urban	34.7 (31.5, 37.8)	37.4 (34.5, 40.2)	45.8 (43.4, 48.3)	47.6 (45.3, 49.8)	47.4 (44.9, 50.0)	51.3 (48.9, 53.7)
Rural	28.9 (25.3, 32.6)	32.9 (30.0, 35.9)	40.8 (38.6, 43.1)	46.5 (44.5, 48.5)	48.0 (45.8, 50.1)	51.0 (49.2, 52.9)

Appendix Table 4. Annual change in standardised prevalence of obesity and overweight defined by Chinese BMI cut-offs (obesity: BMI ≥28 and overweight: ≥24 kg/m^2) in 2004-2010 and in 2010-2018.

	Men			Women			Both		
	**2004-2010**	**2010-2018**	**p***	**2004-2010**	**2010-2018**	**p***	**2004-2010**	**2010-2018**	**p***
**% change (& 95%CI) in obesity prevalence**									
Overall	12.5 (9.3, 16.0)	5.5 (4.2, 6.9)	0.0002	6.2 (3.1, 9.6)	3.0 (1.7, 4.3)	0.0496	9.1 (6.1, 12.4)	4.3 (3.1, 5.6)	0.0045
Urban	12.6 (8.4, 17.3)	4.9 (3.0, 6.8)	0.0015	6.5 (2.1, 11.4)	1.4 (-0.5, 3.3)	0.0312	9.6 (5.6, 14.0)	3.4 (1.7, 5.1)	0.0072
Rural	12.6 (8.0, 17.6)	5.5 (3.7, 7.3)	0.0054	6.1 (1.8, 10.8)	4.1 (2.4, 5.9)	0.2366	8.9 (4.8, 13.6)	4.8 (3.2, 6.4)	0.0536
**% change (& 95%CI) in overweight prevalence**									
Overall	7.3 (5.4, 9.2)	2.8 (2.1, 3.5)	<0.0001	3.7 (2.1, 5.4)	1.7 (1.0, 2.5)	0.0299	5.5 (3.8, 7.2)	2.3 (1.7, 2.9)	0.0006
Urban	5.8 (3.8, 7.9)	2.0 (1.1, 3.0)	0.0014	3.7 (1.7, 5.7)	0.7 (-0.4, 1.8)	0.0126	4.8 (3.0, 6.7)	1.4 (0.5, 2.3)	0.0022
Rural	8.5 (5.8, 11.4)	3.1 (2.2, 4.0)	0.0001	3.7 (1.4, 6.2)	2.6 (1.6, 3.5)	0.2132	5.9 (3.6, 8.5)	2.8 (2.0, 3.7)	0.0142

* p-value for difference in annual changes for 2004-2010 versus 2010-2018.
